# Synthesis, molecular docking analysis, molecular dynamic simulation, ADMET, DFT, and drug likeness studies: Novel Indeno[1,2-*b*]pyrrol-4(1*H*)-one as SARS-CoV-2 main protease inhibitors

**DOI:** 10.1371/journal.pone.0299301

**Published:** 2024-03-22

**Authors:** Davood Gheidari, Morteza Mehrdad, Mohammad Bayat

**Affiliations:** 1 Faculty of Science, Department of Chemistry, University of Guilan, Rasht, Iran; 2 Faculty of Science, Department of Chemistry, Imam Khomeini International University, Qazvin, Iran; Ahram Canadian University, EGYPT

## Abstract

**Background:**

The COVID-19 pandemic began in 2019 as a result of the advent of a novel coronavirus, SARS-CoV-2. At present, there are a limited number of approved antiviral agents for the treatment of COVID-19. Remdesivir, Molnupiravir, and Paxlovid have been approved by the FDA to treat COVID-19 infections. Research has shown that the main protease enzyme (M^pro^) of SARS-CoV-2 plays a crucial role in the enzymatic processing of viral polyproteins. This makes M^pro^ an interesting therapeutic target for combating infections caused by emerging coronaviruses.

**Methods:**

The pharmacological effects of pyrroles and their derivatives have a wide range of applications. In our study, we focused on synthesizing nine novel derivatives of 2-arylamino-dihydro-indeno[1,2-*b*] pyrrol-4(1*H*)-one, with a particular emphasis on their antiviral properties. Using in *silico* studies involving molecular docking and DFT analyses in the gas phase using the B3LYP/6-31++G(d,p) basis set, we studied these compounds with respect to their interactions with the M^pro^ of SARS-CoV-2. The results of the docking analysis revealed that the synthesized compounds exhibited favorable inhibitory effects. Notably, compound **5f** demonstrated the highest effectiveness against the target protein. Furthermore, the pharmacokinetic and drug-like properties of the synthesized derivatives of 2-arylamino-dihydroindeno[1,2-*b*] pyrrol-4(1*H*)-one indicated their potential as promising candidates for further development as inhibitors targeting SARS-CoV-2. However, it is imperative to determine the in *vitro* efficacy of these compounds through comprehensive biochemical and structural analyses.

## 1. Introduction

**Fig 1:**
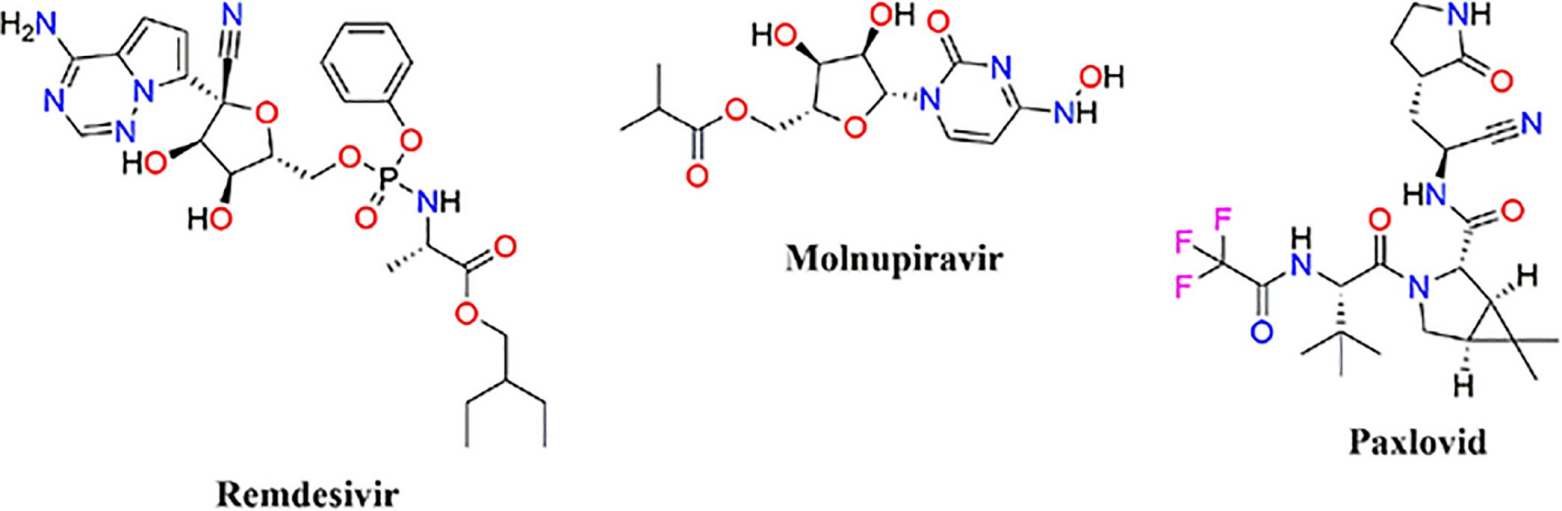
Structure of approved antiviral drugs available for treating patients with COVID-19.

In 2019, an outbreak of respiratory illness with distinct pneumonia symptoms surfaced and swiftly disseminated worldwide, leading the World Health Organization to designate it a pandemic [1]. The disease, which has been named COVID-19, is caused by a previously unknown coronavirus that has been identified as SARS-CoV-2. Contracting SARS-CoV-2 leads to respiratory symptoms resembling mild to moderate influenza, along with severe lung damage and the potential for multiple organ failure, ultimately resulting in death. The ubiquitous spread of SARS-CoV-2 and the constant occurrence of COVID-19 outbreaks pose a significant threat to global public health. There are currently a limited number of antiviral drugs approved for treating COVID-19 patients. Remdesivir, Molnupiravir, and Paxlovid are FDA-approved drugs that have been used for the treatment of COVID-19 [2], Fig 1.

It is anticipated that the drugs will diminish hospitalizations and fatalities linked to COVID-19. Nevertheless, as the pandemic progressed, new SARS-CoV-2 strains like Delta and Omicron emerged. Analyses indicated the Delta variant confers heightened disease severity relative to the ancestral strain, while Omicron displays moderately elevated transmissibility. The recently discovered mutations exhibit genomic anomalies that might possibly result in atypical epidemiological characteristics and the capacity to elude the immune system in the SARS-CoV-2 virus. This might potentially impede the efficacy of the methods of diagnosis and therapy and perhaps instigate further outbreaks [3]. The SARS-CoV-2 virus’s different strains are principally characterized by alterations in the spike protein of the virus. Nonetheless, there is still the potential for mutations in other components of the virus. A recent research has shown that the spike protein and chymotrypsin-like protease (3CL^pro^), also known as the M^pro^, have a vital function in the replication and transcription of the virus. Therefore, they are a desirable target for the creation of therapeutic therapies against SARS-CoV-2 [4]. Consequently, drugs that impede the functioning of this protease have the potential to stop viral replication. *N*-heterocyclic scaffolds are crucial in the study and advancement of drugs since they form the primary structural element of many physiologically active compounds. Their capability to interact with various cellular systems in living organisms has greatly contributed to their versatility. These compounds possess the capacity to disrupt a host factor involved in the replication process, impede the generation of new viral proteases, suppress an enzyme generated by the virus, or obstruct the entrance of the virus into host cells in patients [5–8]. Considering these factors and as part of our ongoing research to develop novel and bioactive *N*-heterocycles, we focused on synthesizing a new series of dihydroindeno[1,2-*b*]pyrrol-4(1*H*)-one derivatives. The aim of our study was to demonstrate the antiviral activity of these compounds against the M^pro^ of SARS-CoV-2 by using in *silico* methods. In fact, studies show that the gradual progress of computer hardware and software technologies has a vital role in reducing costs and facilitating the discovery of new small molecules. The use of computer-aided drug design methods allows for the effective usage of prospective therapeutic targets found by genomic and proteomic projects, therefore making the drug discovery process more efficient. In the field of drug design, numerous advanced in *silico* methods, including docking, molecular dynamics simulation, and a combination of varied sophisticated methodologies, have been used in several studies [9–11].

## 2. Results and discussion

### Chemistry

**Fig 2:**
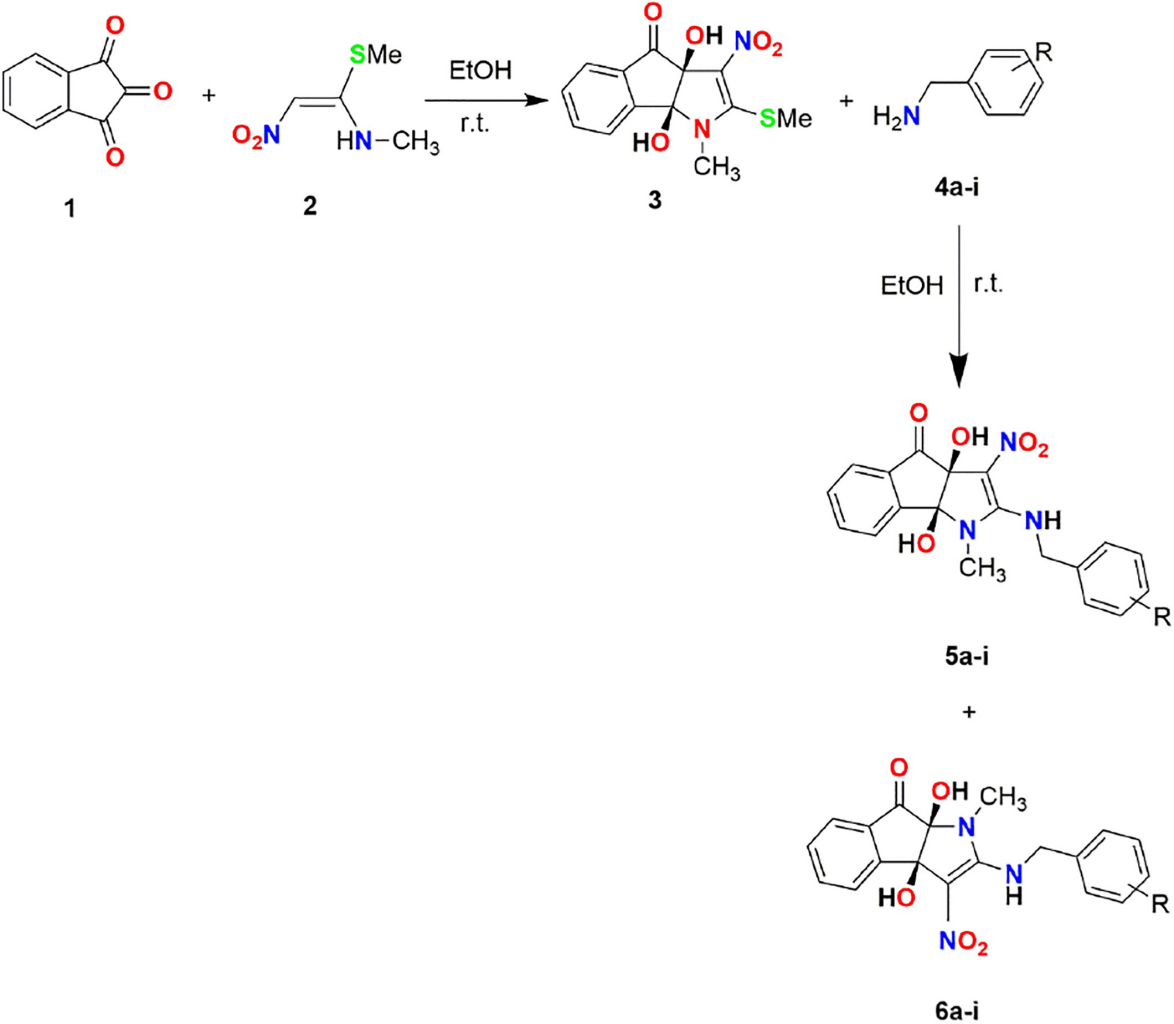
Synthesizing substituted 2-arylamino-dihydroindeno[1,2-*b*]pyrrol-4(1*H*)-one (5a-i) and 2-arylamino-dihydroindeno[2,1-*b*]pyrrol-8(1*H*)-one (6a-i).

**Fig 3:**
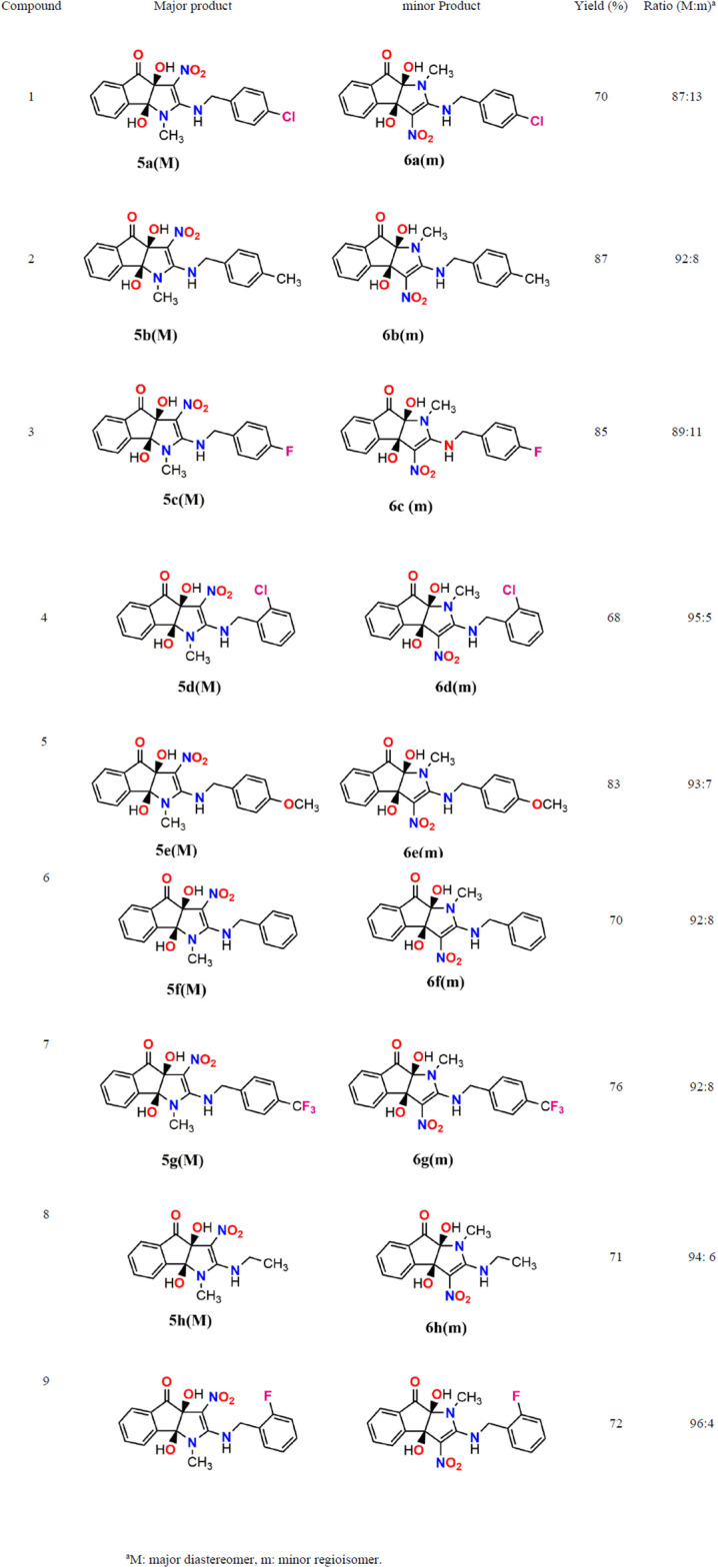
Structures and yields of compounds (5a–i).

To produce the desired compounds, the synthesis process is initiated by subjecting (*E*)-*N*-methyl-1-(methylthio)-2-nitroethenamine and ninhydrin to a reaction in ethanol at ambient temperature. Subsequently, aliphatic amines were added to the product. The reaction has been carried out for one hour, resulting in the formation of the desired 2-arylamino-dihydroindeno[1,2-*b*]pyrrol-4(1*H*)-one (**5a-i**) products with satisfactory yields ranging from 68% to 87%. The identification of compounds (**5a-i**) was accomplished by analyzing their Mass, IR, ^1^H NMR, and ^13^C NMR spectroscopy data, leading to the determination of their structures. The crude product’s ^1^H and ^13^C NMR spectra revealed the presence of two regioisomers. The NMR data for each isomer may be obtained from the mixture of the two isomers, as shown in Fig 2. As an example, the ^1^H and ^13^C NMR of **5a** revealed a key signal for the NH group (δ 9.85 ppm), multiplets for the aromatic groups (*δ* 7.73–7.59 ppm), a doublet for the aromatic groups (*δ* 7.30 ppm), two singlets for the OH group (*δ* 7.22 and 6.37 ppm), a doublet for the aromatic groups (*δ* 7.15 ppm), an ABX system for CH_2_-benzyl groups (*J*_AB_ = 16.0 Hz, *J*_AX_ = 8.0 Hz, *J*_BX_ = 8.0 Hz, *δ*_*A*_ = 4.83 ppm, *δ*_*B*_ = 4.76 ppm) that are diastereotopic, one singlet for the methyl group (*δ* 3.35 ppm) for the major regioisomers (87%); and also one multiplet for the NH group (*δ* 9.46 ppm), multiplets for aromatic and OH groups (*δ* 7.86–7.70 ppm), two doublets for the aromatic groups (*δ* 7.43 and 7.34 ppm), one singlet for the OH group (*δ* 6.51 ppm), two AX systems for the CH_2_-benzyl groups as diastereotopic protons (*δ* 5.39 and 5.19,Δ*δ* = 0.20, ^2^*J*_AX_ = 16.0 Hz), and one singlet for the methyl group (*δ* 2.55 ppm) for the minor regioisomers (13%). The ^1^H-decoupled ^13^C NMR spectra of compound **5a** displayed 16 distinct signals that corresponded with the suggested structure. The mass spectrum of **5a** exhibited a molecular-ion peak at *m/z* 354.1, which corresponds with the suggested structure. The IR spectra displayed absorption bands at 3472, 3352, and 3218 cm^-1^, corresponding to the stretching frequencies of the OH and NH groups. Furthermore, the absorption bands observed at 1733, 1620, 1542, and 1388 cm-1 can correspond to the C = O, NC = C, and NO_2_ functional groups, respectively. Fig 3 displays the complete structures of the synthesized compounds.

**Fig 4:**
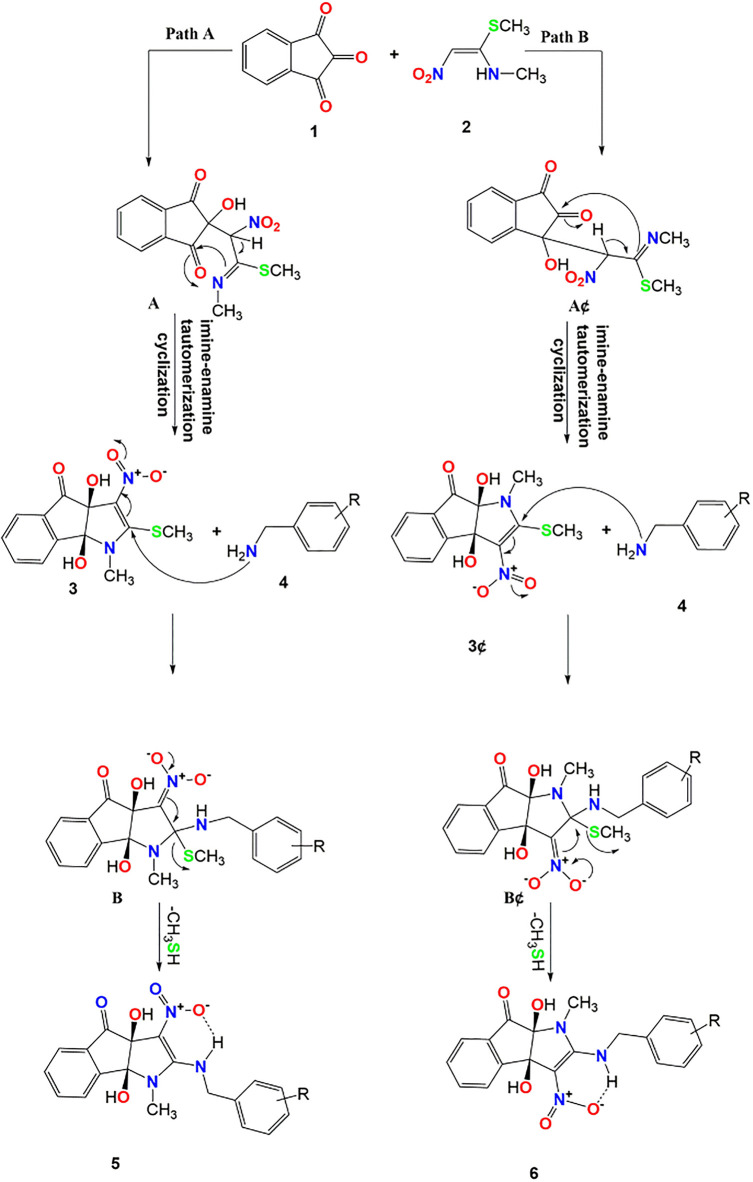
Suggested process for producing compounds 5 and 6.

The mechanism depicted in Fig 4 provides a plausible explanation for synthesizing 2-arylamino-dihydroindeno[1,2-*b*]pyrrol-4(1*H*)-one **5** and 2-arylamino-dihydroindeno[2,1-*b*]pyrrol-8(1*H*)-one **6**. By focusing on the core of ninhydrin, the reaction between the carbonyl group of ninhydrin in positions 1 and 2 and (*E*)-*N*-methyl-1-(methylthio)-2-nitroethenamine results in two products. Indeed, this reaction lacks regioselectivity since the formation of the other regioisomer occurred throughout the process. Following path A as shown in Fig 4, the initial step includes the nucleophilic carbon of enamine 2 reacting with the carbonyl group at position 2 of ninhydrin, resulting in the generation of imine **A** [12,13]. The resultant intermediate **A** undergoes an intramolecular cyclization and an imine-enamine tautomerization to produce diol **3**. Subsequently, a nucleophilic substitution occurs at the C-2 position of dihydroindeno[1,2-*b*]pyrrol-4(3a*H*)-ones **3** with the aliphatic amine **4**. This reaction leads to the formation of intermediate **B**. Following that, the elimination of methanethiol *via* intermediate **B** results in the production of the final product **5**. The aliphatic amine **4** undergoes a nucleophilic substitution reaction on C-2 of dihydroindeno[1,2-*b*]pyrrol-4(3a*H*)-ones **3**, resulting in the formation of intermediate **B**. This intermediate then eliminates methanethiol to generate the final product **5**. Another route is path B in Fig 4, where imine **A’** is produced when enamine engages in a nucleophilic attack on the carbonyl group in position 1 of ninhydrin. The intermediate **A’** undergoes an imine-enamine tautomerization and an intramolecular cyclization to produce diol **3’**. The desired product **6** is obtained *via* the nucleophilic substitution reaction of aliphatic amine **4** on C-2 of dihydroindeno[2,1-*b*]pyrrol-8(1*H*)-one **3’**, resulting in the elimination of methanethiol. The presence of an electron-withdrawing nitro group at position 3 of the pyrrole ring allows for the acceptance of this procedure while also contributing to the resultant structure’s stability via intramolecular hydrogen bonding. Notably, the central C = O, surrounded by two neighboring C = O groups, is likely to possess a higher electrophilic nature. As a result, the formation of compound **A** as the primary product is expected to be favored over the production of compound **A’**.

### Quantum chemistry through density functional theory calculation

All the selected compounds **(5a–i)** have been optimized first under gas phase conditions using the B3LYP/6-31++G(d,p) basis set, and their values are displayed in Table 1.

**Table 1:** Geometric parameters of the compounds (5a–i).

S.no.	Compound	Gas phase		
		Optimization energy (hartree)	Polarizability (α) (a.u.)	Dipole moment (Debye)
1	**5a**	-1734.19	285.36	8.888
2	**5b**	-1313.92	284.68	10.436
3	**5c**	-1373.84	270.01	8.885
4	**5d**	-1734.19	283.60	8.145
5	**5e**	-1389.12	291.14	10.707
6	**5f**	-1274.60	269.76	9.983
7	**5g**	-1611.65	284.54	7.717
8	**5h**	-1082.85	211.98	9.615
9	**5i**	-1373.84	267.80	9.912

Upon minimizing the energy gradient and optimizing the molecular geometries of the selected compounds, no imaginary frequencies were detected. This suggests that all structures correspond to genuine local minima in the potential energy domain. The optimized structures of these compounds are shown in the Supplementary File. The analysis of molecular orbitals (MO) is of outstanding importance in quantum chemistry and enables a comprehensive understanding of chemical behavior. The most important molecular orbitals within a compound include the highest occupied molecular orbital (HOMO) and the lowest unoccupied molecular orbital (LUMO). These orbitals are used to elucidate various chemical properties, including reactivity, stability, and kinetics. The Supplementary File contains the FMO orbitals for the synthesized compounds. The parameter of hardness (*η*) serves as a measure to determine the relative hardness or softness of a molecule. The reactivity of a molecule is enhanced as its softness increases. Electronegativity (*X*) is a fundamental property that quantifies an element’s ability to attract electrons. All these properties were estimated at. The compound **5d** exhibits the smallest HOMO–LUMO energy gap value 3.9638 eV, indicating high chemical reactivity. It also demonstrates a low hardness value 1.9819 and is the softest molecule among all compounds. Additionally, compound **5d** possesses the highest electronegativity value 5.4594, indicating strong electron-attracting capability and making it a better electrophile compared to other compounds. Following **5d**, compounds **5e** and **5b** also display notable reactivity, with energy gap values of 4.0577 eV and 4.0643 eV, respectively. The highest polarizability is related to **5e**. After **5e**, compounds **5g**, **5b**, and **5a** showed remarkable polarizability with values of 284.54, 284.68, and 285.36, respectively. Table 2 illustrates the energetic parameters of the compounds **(5a–i)**.

**Table 2:** Energetic parameters of the compounds (5a–i).

Compound	*E*_*HOMO*_ (eV)	*E*_*LUMO*_ (eV)	ΔE _*gap*_(eV)	Hardness(*η*)	Softness (*S*)	Electronegativity(*X*)	Electrophilicity(*ψ*)
**5a**	-6.3752	-2.2530	4.1222	2.0611	0.2425	4.3141	4.5149
**5b**	-6.1641	-2.0998	4.0643	2.0321	0.2460	4.1319	4.2006
**5c**	-6.3598	-2.2345	4.1253	2.0626	0.2424	4.2971	4.4761
**5d**	-6.2822	-2.31831	3.9638	1.9819	0.2522	5.4594	7.5193
**5e**	-6.1464	-2.0887	4.0577	2.02885	0.2464	4.1175	4.1781
**5f**	-6.1962	-2.1219	4.0743	2.03715	0.2454	4.15905	4.2455
**5g**	-6.3537	-2.2351	4.1186	2.0593	0.2428	4.2944	4.4777
**5h**	-6.2484	-2.1548	4.0936	2.0468	0.2025	4.2016	4.3124
**5i**	-6.2005	-2.1796	4.0209	2.0104	0.2487	4.1900	4.3663

### Molecular docking studies

**Fig 5:**
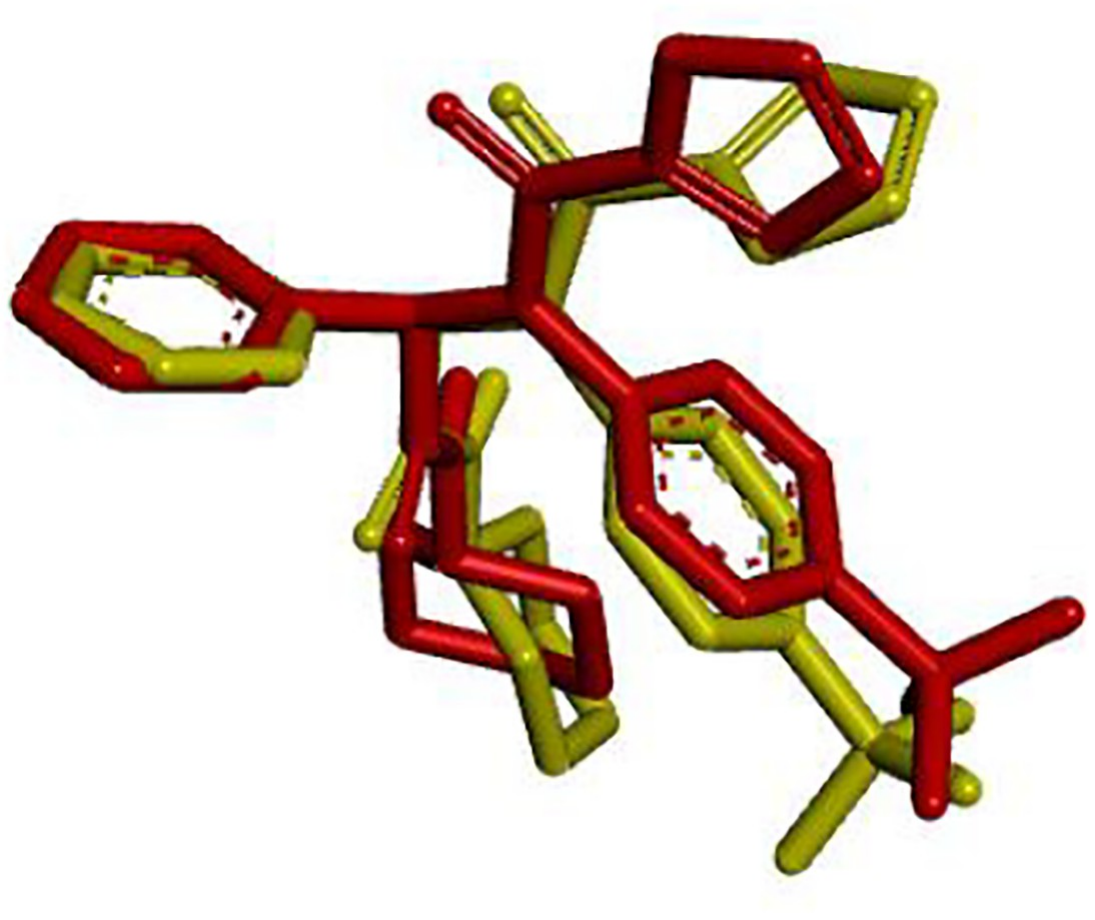
Superimposition of the docked ligand (red) and the original ligand (green).

Molecular docking was used to assess the binding mechanisms of synthesized compounds with SARS-CoV-2 M^pro^. The targeting of this protease is important to develop therapeutic approaches against SARS-CoV-2. These targets were selected based on their critical functions in viral protein production; hence, targeting these proteins may provide benefits in virus killing. The crystal structure of the M^pro^ protein identified by the PDB ID: 6W63, has been acquired from the RCSB database. The docking procedure was validated by re-docking the co-crystal ligand into the active pocket of the SARS-CoV-2 M^pro^ protein. The observed root-mean-square deviation (RMSD) value of 1.18 Å indicates a high level of reliability for the docking procedure [14] (Fig 5).

**Fig 6:**
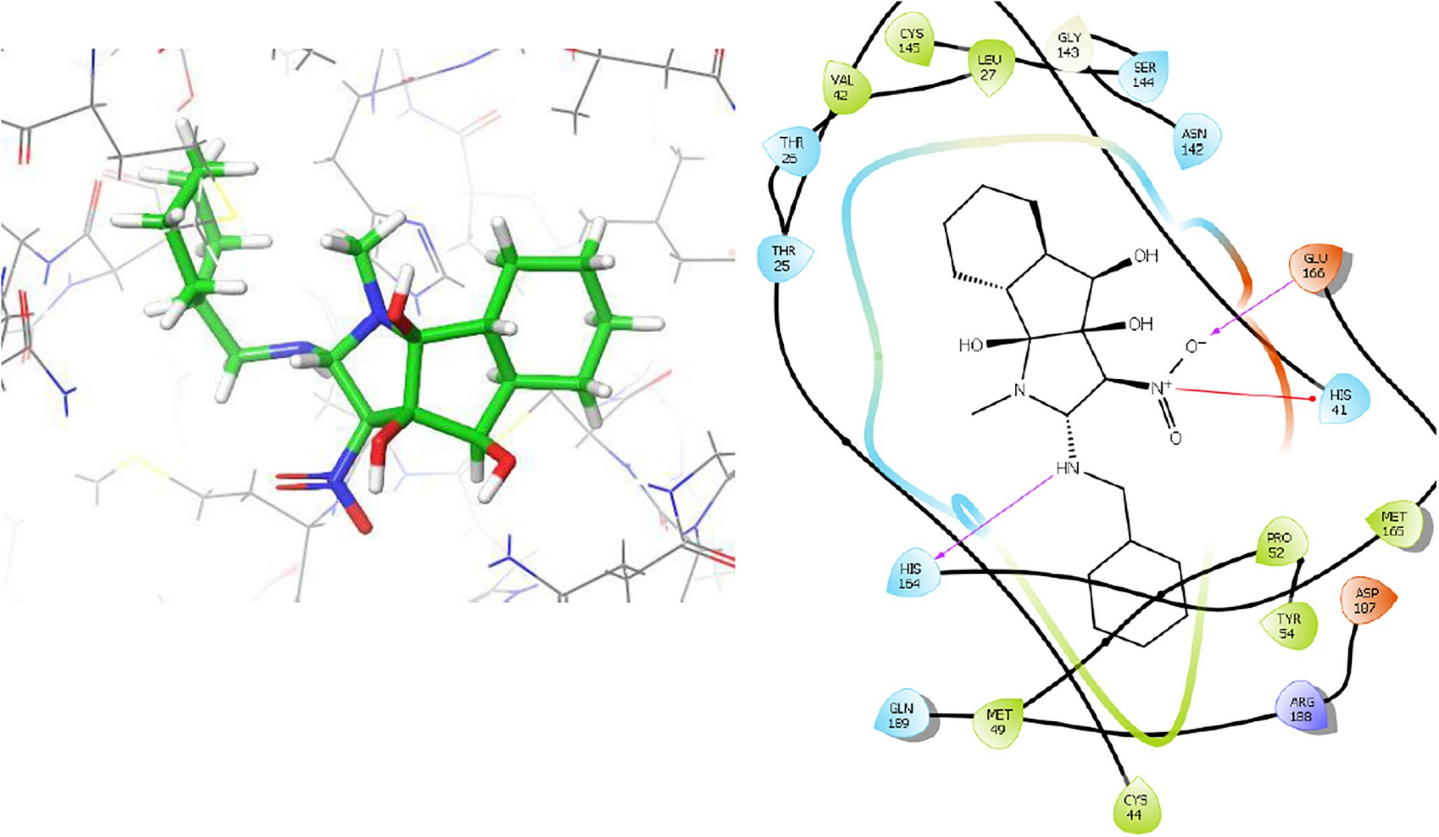
The 3D and 2D binding modes of 5f in the active pocket of SARS-CoV-2 M^pro^.

The key amino acid residues in the catalytic site were investigated in docking studies with potent indeno[1,2-*b*]pyrrol-4(1*H*)-one derivatives. The results indicated that a considerable portion of the compounds demonstrated substantial binding scores, and among them, compound **5f** displayed the most favourable binding energy at -7.28 Kcal/mol. The best configuration of **5f** was selected to study both bonding and non-bonding interactions. The intricate 3D and 2D binding interactions of compound **5f** within the active pocket of the SARS-CoV-2 M^pro^ protein are illustrated in Fig 6. The amino acid residues Glu166, His164, Gly143, Met165, Arg188, Pro52, Tyr54, Asp187, Thr25, Val42, Thr26, Ser144, Asn142, Gln189, His41, Cys145, Leu27, Met49, and Cys44 were involved in both bonding and non-bonding interactions with compound **5f**. In summary, compound **5f** exhibits four hydrogen bond interactions, encompassing two conventional hydrogen bonds and two carbon hydrogen bonds. Furthermore, it has been observed that compound **5f** is accountable for establishing five hydrophobic contacts with the amino acids His41, Cys145, Leu27, Met49, and Cys44 at the active site. Moreover, the amino acid residues Arg188, Pro52, Tyr54, Asp187, Thr25, Val42, Thr26, Ser144, Asn142, and Gln189 were identified as participants in the van der Waals interactions with compound **5f**. The docking outcomes of the potent compounds, along with their respective interactions, are delineated in Table 3.

**Table 3:** Docking scores, and interaction of each compounds (5a–i) against SARS-CoV-2 M^pro^.

Compound	Docking scores (kcal/mol)	Interaction residue					
		Hydrogen Bond			Van der walls	Hydrophobic	Others
		H-bonding(distance)	C-bonding	π-Donor H-bonding			
**5a**	-6.73	Gly143(2.86),His164(2.72),Asn142(2.73)			Met165, Cys145, Gln189, Arg188, Tyr154, Pro52, Thr25, Ser144, His163, Glu166, Phe140, Leu141, His172	His41, Cys44,Met49	
**5b**	-7.05	His41(2.76)	His164		Cys44,Cys145,Gly143,His163,Ser144,Asn152,Phe140,His172,Leu141,Glu166,Met165,Gln189,Arg188,Asp187,Tyr154,Pro52	Met49	
**5c**	-6.44	Gln189(2.97),His164(2.43),His41(2.83)	Pro168,Arg188	His41	Asp187,Met49,Cys44,Thr25,Asn142,Gly143,Cys145,Glu166,Leu167,Gln192	Met165,Pro168	Thr190
**5d**	-6.56	Cys145(2.95),Glu166(2.77)	Glu166		Cys44,Tyr54,Asp187,His164,Asn142,Leu167,Gln192,Thr190,Gln189,Pro52	Pro168,Met165,Met49,His41	Arg188
**5e**	-6.77	His164(2.41),Glu166(2.44)	Thr190,Pro168		Pro52,Tyr54,Asp187,Cys145,Arg188,Met165,Leu167,Gln192,Gln189,	His41,Met49,Cys44	
**5f**	-7.28	Glu166(2.37),His164(2.31),Gly143(2.82)	Met165		Arg188,Pro52,Tyr54,Asp187,Thr25,Val42,Thr26,Ser144,Asn142,Gln189	His41,Cys145,Leu27,Met49,Cys44	
**5g**	-6.56	Gln189(2.45),Gly143(2.71)	Gln189,Arg188	His41	Tyr54,Thr45,Ser46,Thr24,Thr25,Thr26,Leu27,His164,Cys145,Glu166,Met165,Asp187	His41	Asn142,Met49,Arg188
**5h**	-6.32		His41		Thr25, Thr45, Ser46, Pro52, Asp187, Tyr54, Arg188, Thr190, Gln192, Gln189, Glu166, Cys145, His164	Met165,Cys44,Met49	
**5i**	-6.29	His41(2.67),Cys145(3.10)	Arg188,Glu166		Thr25,Met49,Asp187,Gln189,Thr190,Ala191,Gln192,Leu167,Met165,His164,Asn142	Pro168	

### Molecular dynamics simulation

**Fig 7:**
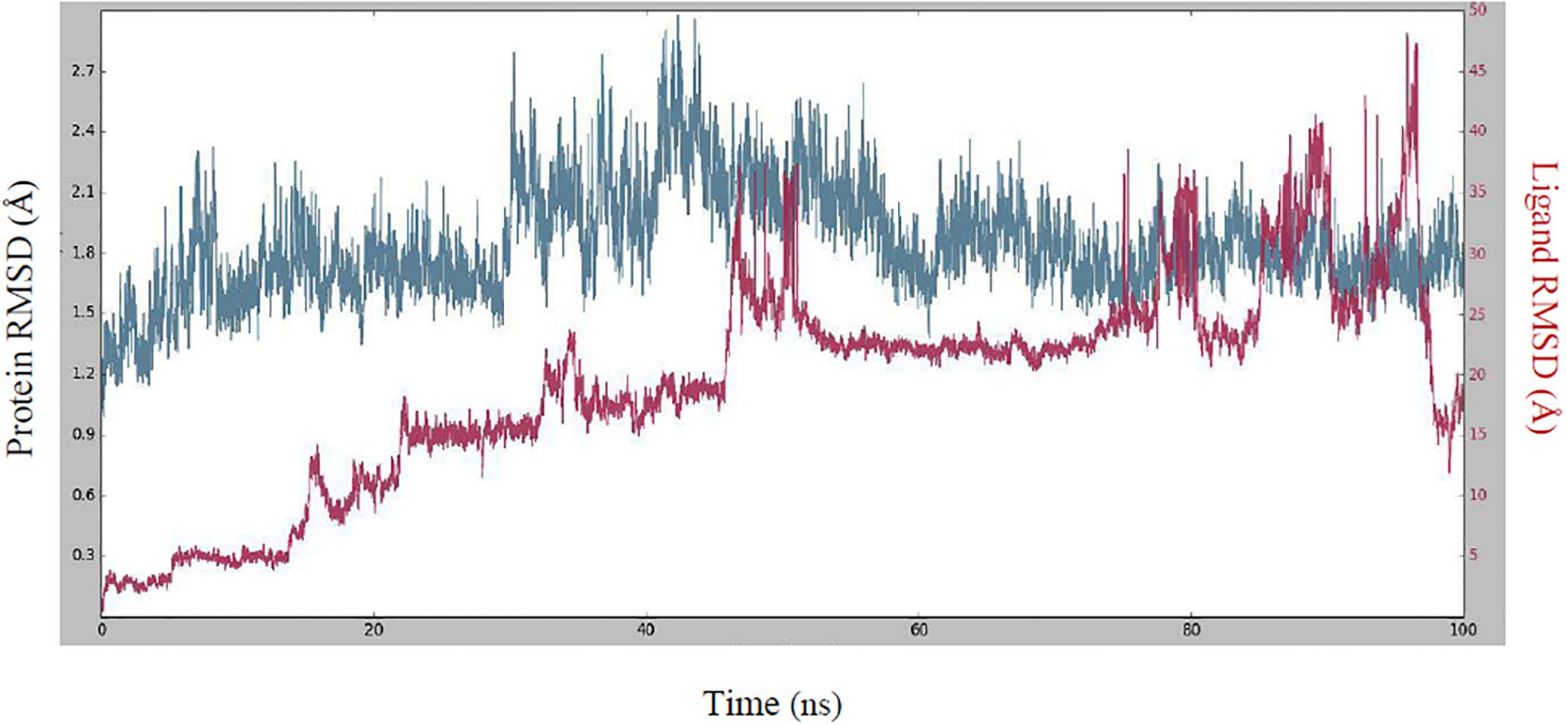
RMSD values of the protein and the ligand during a MD simulation.

**Fig 8:**
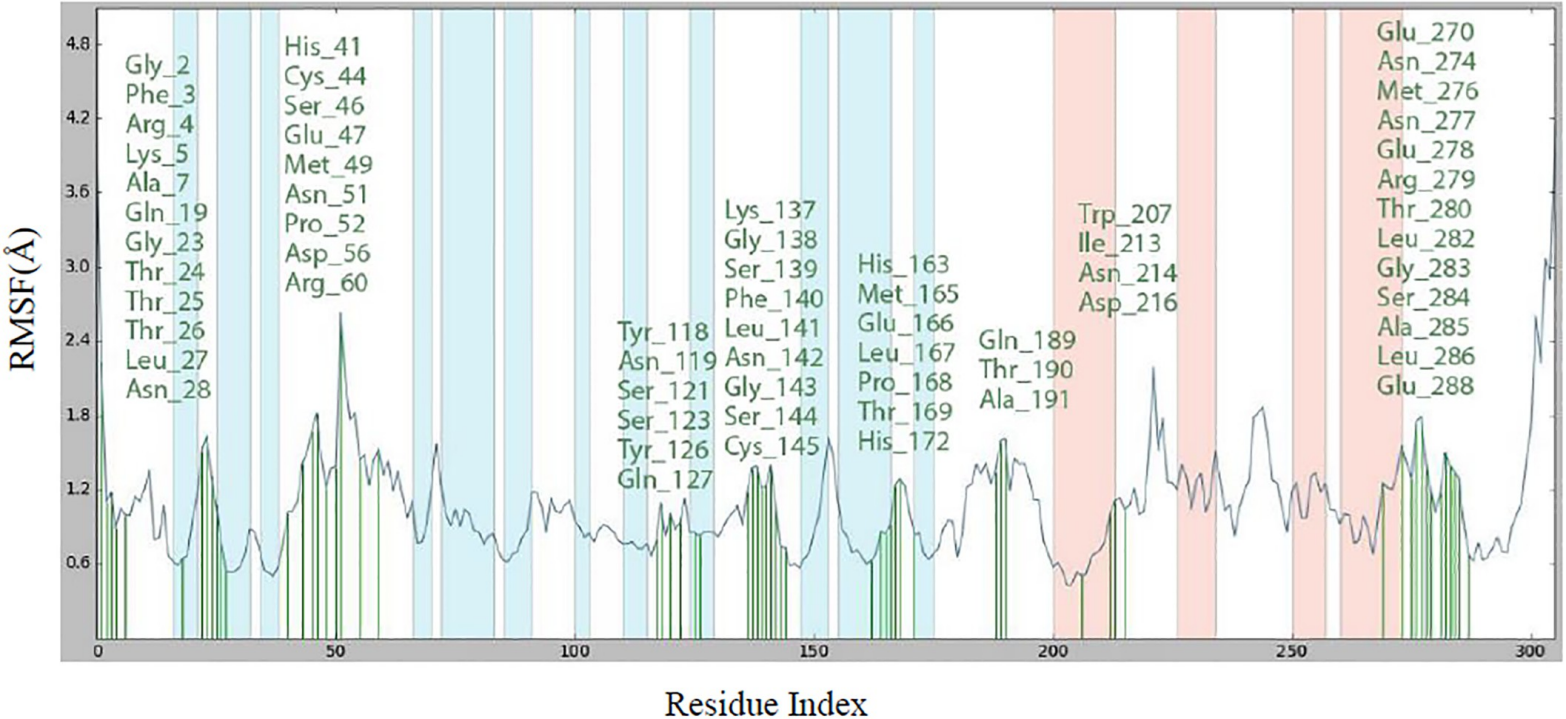
RMSF plot for Cα of CDK2 residues in compound 5f-SARS-CoV-2 M^pro^ complex.

MD simulation of 100 ns was performed for the best protein-ligand complex. Fig 7 illustrates the importance of the RMSD of both the protein (left Y-axis) and the ligand (right Y-axis) as crucial indicators for verifying the accuracy of the docking geometry. The RMSD of the protein provides detailed insight into its structural conformation during the simulation, while the RMSD of the ligand serves as an indicator of the stability of the ligand in the binding pocket of the protein. The RMSD value fluctuates irregularly at the beginning and reaches stability at 53 ns, but loses stability after 78 ns. The RMSD value of the protein is between 0.95 and 2.9 Å throughout the simulation, which is considered an acceptable range for a small globular protein. The RMSD value of the Lig ft Prot was much lower than the RMSD value of the protein during the simulation time, indicating that the ligand was within the initial binding site during the simulation. The RMSF diagram for the ligand–SARS-CoV-2 M^pro^ complex can be used to assess the stability of the individual interactions. The comprehensive analysis of the interaction between compound **5f** and the residues of the SARS-CoV-2 M^pro^ binding site is shown in Fig 8. The residues that interact with compound **5f** are as follows: Gly2, Phe3, Arg4, Lys5, Ala7, Gln19, Gly23, Thr24, Thr25, Thr26, Leu27, Asn28, His41, Cys44, Ser46, Glu47, Met49, Asn51, Pro52, Asp56,Arg60,Tyr118,Asn119,Ser121,Ser123,Tyr126,Gln127,Lys137,Gly138,Ser139,Phe140,Leu141,Asn142,Gly143,Ser144,Cys145,His163,Met165,Glu166,Leu167,Pro168,Thr169,His172,Gln189,Thr190,Ala191,Trp207,Ile213,Asn214,Asp216,Glu270,Asn274,Met276,Asn277,Glu278,Arg279,Thr280,Leu282,Gly283,Ser284,Ala285,Leu286,and Glu288. The residue interacting with compound **5f** is depicted in green. The visualization indicates the protein’s secondary structures-helices and β-strands represented by orange and blue bands, respectively. RMSF values for the residues within the binding site were calculated to be below 3 Å.

**Fig 9:**
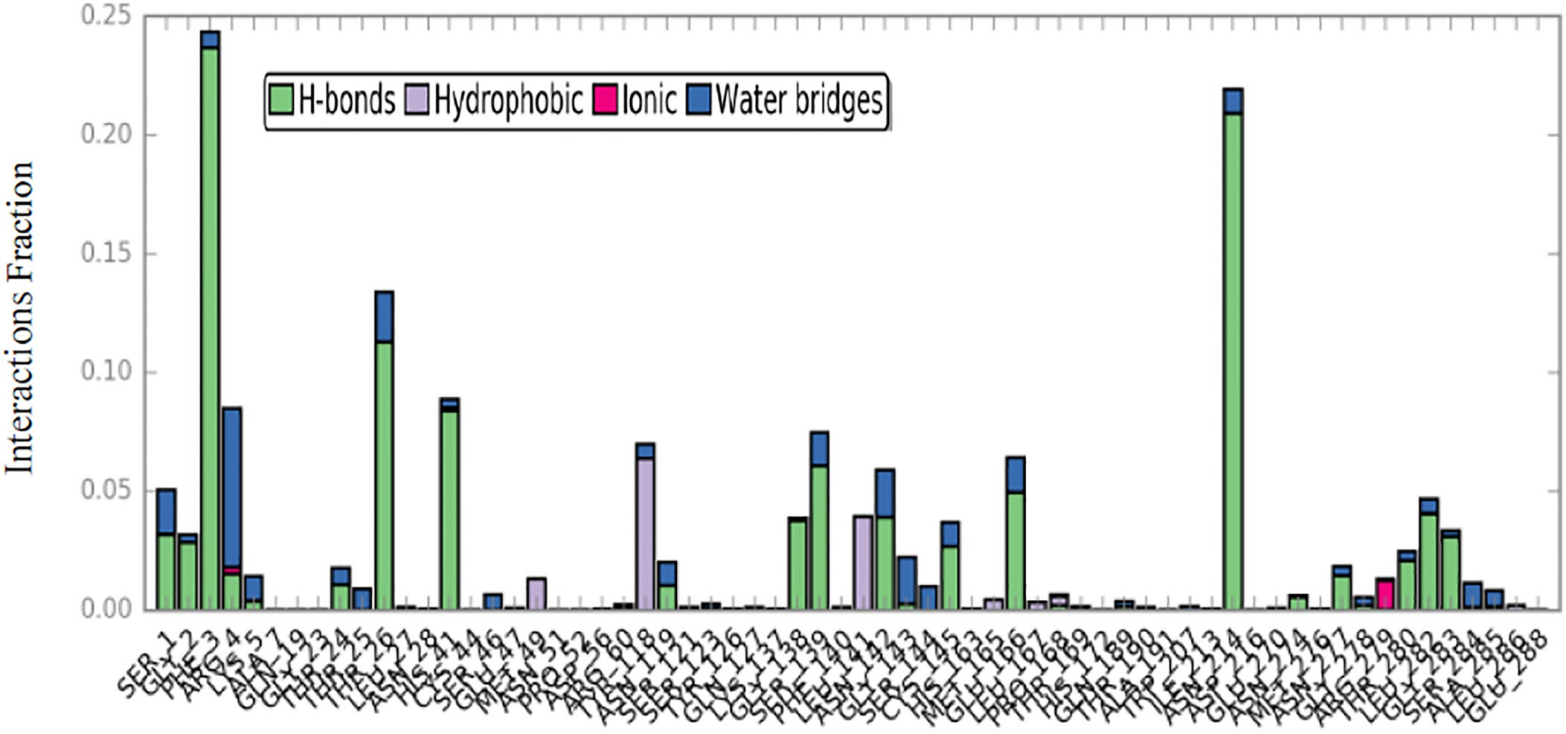
Protein–ligand contacts during simulation time.

Throughout the simulation, the interactions between the ligand and protein can be consistently monitored. These interactions encompass various categories, such as hydrogen bonds, hydrophobic interactions, ionic bonds, and water bridges. Fig 9 shows the histograms of the interaction fractions of the ligand with each of the key residues of the protein during the simulation time of 100 ns. The hydrogen bonding interactions were shown in green columns, the hydrophobic interactions in purple columns, and the water-bridged hydrogen bonding interactions in blue columns. The most frequent interactions with Phe30 and Asn214 were observed during the entire simulation time.

**Fig 10:**
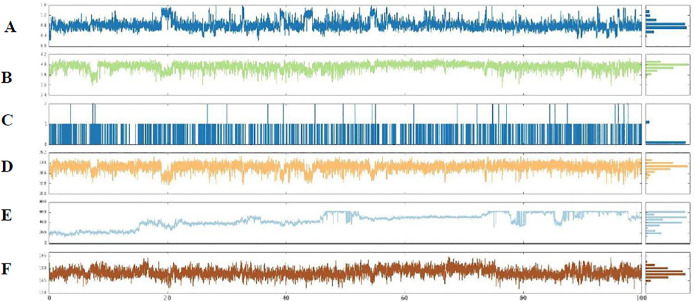
(A) RMSD, (B) rGyr, (C) intraHB, (D) MolSA, (E) SASA, and (F) PSA of the ligand–protein complex calculated during the 100 ns of MD simulation.

Fig 10 illustrates the properties of compound **5f** as determined through a 100-ns MD simulation. Six ligand properties were evaluated: ligand RMSD, radius of gyration (rGyr), intramolecular hydrogen bonding (intra-HB), molecular surface area (MolSA), solvent-accessible surface area (SASA), and polar surface area (PSA). The RMSD of **5f** ranged from 0.2 to 1.6 Å, with an equilibrium around 0.8 Å. The rGyr was within the range of 3.5 to 4.2 Å, with an equilibrium at about 4 Å. During the entire MD simulation, the intra-HB remained constantly high. MolSA fluctuated in the range of 328–350 Å^2^, with an equilibrium value of about 345 Å^2^. SASA exhibited significant fluctuations, ranging from about 200 to 600 Å^2^ between 17 and 50 ns and then stabilizing at about 400 Å^2^. PSA fluctuated in the range of 157–190 Å^2^, with an equilibrium value around 178 Å^2^.

### Drug-likeness prediction

Drug likeness refers to the extent of similarity between certain compounds and established drugs. This assessment involves a nuanced balance of molecular and structural properties. Drug similarity assessment is based on various molecular properties such as hydrophobicity, electronic distribution, hydrogen bonding, molecular weight, pharmacophores moiety, bioavailability, reactivity, toxicity, and metabolic stability [15]. The Lipinski rule is a widely used method for assessing the solubility and permeability of compounds and thus predicting their suitability as potential drug candidates. According to this rule, compounds that violate Lipinski’s rule of five are more likely to have insufficient absorption or permeation. Using the online web server SwissADME [16], our derivatives were analyzed. All of the synthesized compounds **(5a–i)** do not break the Lipinski rule because their values are within the normal range and their absorption. Also, these compounds **(5a–i)** are in the best part of the physiochemical space, so they could be thought of as lead compounds. The pharmacokinetic factors showed that the tested compounds **(5a–i)** are well absorbed by the gastrointestinal system (GI) after being taken by mouth and that P-glycoprotein (P-gp) could move them out of the body. In pharmaceutical chemistry, the pan-assay interference substances (PAINS) structural alerts have been utilized to figure out which parts of the structure are unstable, reactive, and poisonous [17,18]. All of the compounds **(5a–i)** have no alerts in the PAINS descriptions, which is another sign that they could be good drug prospects. The Synthetic Accessibility Score (SA score) serves as a benchmark for assessing the feasibility of synthesizing drug-like molecules. All compounds were found to have a favorable SA score, indicating that they can be easily synthesized, Table 4.

**Table 4:** Physicochemical, pharmacokinetics, and medicinal chemistry properties of the compounds (5a–j).

	MW (g/mol)	HBA	HBD	TPSA	Consensus Log Po/w *	MR	GI Absorption	BBB Permeant	P-gp Substrate	Lipinski	Pfizer	PAINS (alert)	Bioavailability Score	Synthetic accessibility score
				(Å^2^)										
**5a**	401.8	5	3	118.62	1.55	105.59	High	No	Yes	Yes	Yes	0	0.55	4.27
**5b**	381.38	5	3	118.62	1.21	105.54	High	No	Yes	Yes	Yes	0	0.55	4.38
**5c**	385.35	6	3	118.62	1.17	100.54	High	No	Yes	Yes	Yes	0	0.55	4.27
**5d**	401.80	5	3	118.62	1.59	105.59	High	No	Yes	Yes	Yes	0	0.55	4.28
**5e**	397.38	6	3	127.85	0.97	107.07	High	No	Yes	Yes	Yes	0	0.55	4.35
**5f**	367.36	5	3	118.62	0.90	100.58	High	No	Yes	Yes	Yes	0	0.55	4.27
**5g**	435.35	8	3	118.62	2.04	105.58	High	No	Yes	Yes	Yes	0	0.55	4.40
**5h**	305.29	5	3	118.62	0.04	80.90	High	No	Yes	Yes	Yes	0	0.55	4.02
**5i**	479.43	6	3	118.62	1.29	100.54	High	No	Yes	Yes	Yes	0	0.55	4.30

MW: Molecular Weight; HBA: Num. H-Bond Acceptors; HBD: Num. H-Bond Donors; MR: Molar Refractivity; TPSA: Topological Polar Surface Area; P-M: Poor-Moderate; P: Poor; GI: Gastrointestinal; BBB: Blood–Brain Barrier; P-gp: P Glycoprotein.

### ADMET properties

During the development of novel therapeutic drugs, pharmacological and toxicological knowledge is of the utmost importance. This knowledge not only decreases the duration of drug development but also enhances the success rate. ADMET indices, which encompass Absorption, Distribution, Metabolism, Excretion, and Toxicity, are commonly employed to evaluate the nature of a substance. In the case of dihydroindeno[1,2-*b*]pyrrol-4(1*H*)-one derivatives, these parameters are determined utilizing the ADMET Lab 2.0 [19]. CaCo-2 cells derived from human colonic epithelial cells serve as a common model for the assessment of drug uptake in the human gut. Also, Madin Darby Canine Kidney (MDCK) cells are valuable for the assessment of the rapid permeability of drug molecules due to their shortened growth cycle compared to CaCo-2 cells [20]. The CaCo-2 cell permeability results for our synthesized compounds fell within an acceptable range, suggesting that these compounds possess exceptional intestinal absorption. Moreover, all compounds displayed positive MDCK cell permeability, indicating a higher probability of elimination through kidney cells. The results indicated that all compounds exhibited characteristics suggestive of being both PGP-substrates and PGP-inhibitors in relation to plasma glycoprotein (PGP) interactions. Based on the human intestinal absorption (HIA) values, it can be inferred that all compounds have a favorable likelihood of being absorbed through the intestinal membrane. Compounds exhibiting CBrain/CBlood values exceeding 1 are classified as having central nervous system (CNS) activity, whereas compounds with CBrain/CBlood values below 1 are classified as CNS inactive. Compounds possessing central nervous system (CNS) activity have the ability to cross the Blood-Brain Barrier (BBB) and result in side effects on the central nervous system [21]. According to the data presented in Table 5, all of our compounds have CBrain/CBlood values below 1, indicating that they are not capable of crossing the BBB. Consequently, our synthesized compounds are devoid of neurotoxicity. The percentage representation of the plasma protein binding model determines the extent to which a compound is strongly bound to blood carrier proteins. The plasma protein binding values for compounds **5a**, **5d**, and **5g** exceed 94%, suggesting that our synthesized derivatives of dihydroindeno[1,2-*b*]pyrrol-4(1*H*)-one possess sufficient bioavailability and are unlikely to exhibit strong binding to blood carrier proteins.

**Table 5:** ADMET profile of the compounds (5a–j).

Absorption and Distribution								
	Caco-2 Permeability	MDCK Permeability	PGP-Inhibitor	*p*-Glycoprotein substrate (PGPsubstrate)	Human intestinal absorption (HIA)	Plasma protein binding (PPB)	Volume of distribution (VD)	Blood brain barrier (BBB) penetration (c.brain/ c.blood)
Mode								
**5a**	-4.687	0.00015	---0.001	---0.052	---0.007	95.46	1.102	-0.44
**5b**	-4.651	0.00014	---0.003	--0.233	---0.006	92.84	1.097	-0.316
**5c**	-4.737	0.00015	---0.005	---0.041	---0.006	93.60	0.902	--0.221
**5d**	-4.723	0.00015	---0.002	---0.033	---0.006	94.49	1.127	+0.556
**5e**	-4.763	0.00011	---0.004	---0.062	---0.007	91.34	0.983	-0.427
**5f**	-4.828	0.00017	---0.001	---0.033	---0.01	90.36	0.888	+0.514
**5g**	-4.693	9.1e-05	---0.005	---0.073	---0.006	95.38	1.127	--0.12
**5h**	-4.953	4.3e-05	---0.001	---0.092	--0.172	50.043	1.026	--0.284
**5i**	-4.728	0.000169	---0.002	---0.032	---0.005	93.05	0.883	--0.293

Metabolism											Elimination	
	CYP1A2 inhibitor	CYP1A2 Substrate	CYP2C19 inhibitor	CYP2C19 Substrate	CYP2C9 inhibitor	CYP2C9 Substrate	CYP2D6 inhibitor	CYP2D6 Substrate	CYP3A4 inhibitor	CYP3A4 Substrate	T_1/2_	CL
**5a**	++0.757	--0.29	+++0.921	++0.848	++0.785	---0.064	---0.097	-0.342	++0.876	+++0.931	0.107	3.766
**5b**	+0.601	--0.163	++0.871	++0.862	++0.749	---0.066	---0.017	+0.561	++0.875	+++0.93	0.124	3.993
**5c**	+0.565	--0.187	++0.865	++0.852	+0.687	---0.064	---0.024	+0.513	++0.813	+++0.924	0.085	3.881
**5d**	++0.745	--0.181	+++0.914	++0.836	++0.746	---0.064	---0.041	-0.333	+++0.918	+++0.929	0.097	4.485
**5e**	-0.434	--0.23	++0.878	++0.862	++0.713	---0.074	---0.023	++0.789	++0.899	+++0.927	0.137	5.045
**5f**	+0.556	--0.116	+0.67	++0.852	+0.635	---0.058	---0.01	--0.224	++0.745	+++0.926	0.135	4.032
**5g**	++0.712	--0.152	+++0.929	++0.826	++0.793	---0.06	---0.027	-0.334	++0.883	+++0.918	0.052	3.612
**5h**	-0.327	--0.173	---0.171	++0.856	--0.225	---0.047	---0.002	--0.384	--0.487	+++0.298	2.103	1.064
**5i**	+0.652	--0.139	++0.843	++0.847	+0.67	---0.071	---0.026	-0.459	++0.847	+++0.922	0.084	4.198

	**Toxicity**							
	**AMES toxicity**	**Carcinogenicity**	**Eye corrosion**	**Eye irritation**	**hERG**	**H-HT**	**LD**_**50**_	**Respiratory toxicity**
**5a**	++0.86	+++0.949	---0.003	---0.012	---0.008	--0.202	638.288	++0.867
**5b**	++0.899	+++0.948	---0.003	---0.016	---0.005	--0.252	442.963	++0.864
**5c**	+++0.902	+++0.961	---0.003	---0.013	---0.006	-0.409	523.427	++0.867
**5d**	++0.787	+++0.939	---0.003	---0.015	---0.004	--0.196	587.512	++0.282
**5e**	++0.891	+++0.936	---0.003	---0.015	---0.007	-0.397	450.001	++0.876
**5f**	++0.897	+++0.905	---0.003	---0.018	---0.004	--0.199	409.348	++0.877
**5g**	++0.781	+++0.955	---0.003	---0.012	---0.007	--0.236	198.997	+++0.932
**5h**	++0.854	---0.353	---0.004	---0.031	---0.003	---0.201	459.952	+0.679
**5i**	++0.897	+++0.931	---0.003	---0.015	---0.003	+0.540	504.495	++0.833

Similarly, the volume distribution (VD) findings suggest that compounds **5a**, **5d**, and **5g** are not the most limited to blood components and are therefore uniformly distributed across blood and tissue components. Toxicology research is important in drug design since it helps to identify the adverse effects of novel entities on live organisms. The calculation of toxicity indices for all synthesised dihydroindeno[1,2-*b*]pyrrol-4(1*H*)-one derivatives revealed that all of the compounds are noncarcinogenic. Moreover, the AMES toxicity assessment revealed that the compounds exhibit no potential for toxicity. Consequently, our synthesized compounds are devoid of any toxic effects. In general, all compounds demonstrated an enhanced ADMET profile, with corresponding values provided in Table 5.

## 3. Conclusion

We have effectively synthesized nine novel indeno[1,2-*b*]pyrrol-4(1*H*)-one derivatives and identified them using IR, ^1^H, and ^13^C NMR in this study. The structural geometry was accurately predicted using DFT calculations. Our in *silico* results show that these compounds have an inhibitory effect against SARS-CoV-2 M^pro^. In fact, the results showed that compound **5f** is more potently inhibitory than the other compounds. Moreover, our result showed that the pharmacokinetics of indeno[1,2-*b*]pyrrol-4(1*H*)-one are valuable and provide an informative and promising investigation as effective SARS-CoV-2 M^pro^ inhibitors. Consequently, it hinders the process of viral replication and may serve as a potential therapeutic intervention for combating SARS-CoV-2. Further validation of the chemoinformatics study’s findings would require additional in *vivo* and in *vitro* investigations.

### Experimental

#### General information

All reagents and solvents obtained from Aldrich and Merck Chemical Co. DMSO-*d*_*6*_ were used as solvents for the NMR analyses. Bruker at 400 MHz for ^1^H and 100 MHz for ^13^C was used. The chemical shifts were recorded in parts per million (ppm), and the coupling constants (*J*) were expressed in Hz. Melting points have been determined using an electrothermal 9100 instrument. The Agilent 5975C VL MSD with a triple-axis detector was used to record mass spectra at an ionization potential of 70 eV. The Bruker Tensor 27 spectrometer has been used to analyses IR spectra.

#### General procedure for the synthesis of compounds (5a-i)

Equal amounts of ninhydrin **1** and (*E*)-*N*-methyl-1-(methylthio)-2-nitroethenamine **2** were mixed in 3 mL of ethanol at room temperature for 5 minutes. Product precipitation from the clear solution indicated reaction completion. The precipitate was filtered, washed with 10 mL of water, and dried to yield 3a,8b-dihydroxy-1-methyl-2-(methylthio)-3-nitro-3a,8b-dihydroindeno[1,2-*b*]pyrrol-4(1*H*)-one **3**. Equal amounts of compound **3** and aliphatic amines were produced in 5 mL of ethanol. The mixture was swirled for 1 hour at room temperature. The 2-arylamino-dihydroindeno[1,2-*b*]pyrrol-4(1*H*)-one **(5a-i)** products yielded 68% to 87% after a 1-hour reaction.

**5a:** White solid; m.p 212–214°C; IR (KBr): 3472 (OH), 3352 (OH), 3218 (NH), 1733 (C = O), 1620 (NC = C), 1542 and 1388 cm^-1^ (NO_2_). MS: *m/z* (%) = 354.1 (5) [M-NO_2_]^+^, 195.1 (21), 166 (10), 140 (27), 127 (33), 125 (100), 104 (50), 89.1 (20), 76.1 (40), 56.1(18).

**Major isomer (87%): *2-((4-chlorobenzyl)amino)-3a*,*8b-dihydroxy-1-methyl-3-nitro-3a*,*8b-dihydroindeno[1*,*2-b]pyrrol-4(1H)-one*.**^1^H NMR: δ ppm, δ 9.87 (1H, *t*,^3^*J*_HH_ = 6.0, NH), 7.73–7.59 (4H, *m*, ArH), 7.30 (2H, *d*, ^3^*J*_*HH*_ = 8.0, ArH), 7.22 (1H, *s*, OH), 7.15 (2H, *d*, ^3^*J* = 8.0, ArH), 6.37 (1H, *s*, OH),4.83–4.77 (ABX, 2H, *m*, NCH_2_), 3.35 (3H, s, NCH_3_). ^13^C NMR: δ ppm, 29.1 (N-CH_3_), 45.5 (N-CH_2_), 80.9 (C-OH), 93.8 (C-OH), 123.1 (C-NO_2_), 124.1, 128.1, 128.6, 130.5, 131.9, 133.6, 135.3,136.65, 137.5, 146.8, (Ar), 154.1 (= C-NCH2), 196.1 (C = O).

**Minor isomer (13%):*2-((4-chlorobenzyl)amino)-3a*,*8a-dihydroxy-1-methyl-3-nitro-1*,*8a-dihydroindeno[2*,*1-b]pyrrol-8(3aH)-one*.**^1^H NMR: δ 9.46 (1H, *br m*, NH), 7.86–7.70 (5H, *m*, ArH and OH), 7.43 (2H, *d*, ^3^*J*_*HH*_ = 8.0, ArH), 7.34 (2H, *d*, ^3^*J* = 8.0, ArH), 6.51 (1H, *s*, OH), 5.39 (1H, *d*, ^2^*J*_AX_ = 16.0, CH_2_), 5.19 (1H, *d*, ^2^*J*_AX_ = 16.0, CH_2_), 2.55 (3H, s, NCH_3_).

**5b:** White solid; m.p 185–187°C; IR (KBr): 3458 (OH), 3385 (OH), 3232 (NH), 1730 (C = O), 1617 (NC = C), 1552 and 1382 cm^-1^ (NO_2_). MS: *m/z* (%) = 334.1 (6) [M-NO_2_]^+^, 287.1 (4), 175.1 (25), 159.1 (8),132.1 (25) 120.1 (33), 105.1 (100), 104.1 (66), 76.1 (49), 50.1 (21).

**Major isomer (92%): *3a*,*8b-dihydroxy-1-methyl-2-((4-methylbenzyl)amino)-3-nitro-3a*,*8b-dihydroindeno[1*,*2-b]pyrrol-4(1H)-one*.** ^1^H NMR: δ ppm, δ 9.85 (1H, *t*,^3^*J*_HH_ = 8.0, NH), 7.76–7.60 (5H, *m*, ArH and OH), 7.07–7.02 (4H, *m*, ArH), 6.36 (1H, *s*, OH),4.83–4.74 (ABX, 2H, *m*, NCH_2_), 3.77 (3H, *s*, NCH_3_), 2.26 (3H, s, CH_3_).^13^C NMR: δ ppm, 29.0 (N-CH_3_), 20.6 (CH_3_), 46.0 (N-CH_2_), 80.0 (C-OH), 92.9 (C-OH), 107.6 (C-NO_2_),123.2,124.3,125.8,126.2, 130.5, 133.7,135.2,135.5,136.6,146.8 (Ar), 153.9 (= C-NCH2), 195.1 (C = O).

***Minor isomer (8%)*: *3a*,*8a-dihydroxy-1-methyl-2-((4-methylbenzyl)amino)-3-nitro-3a*,*8a-dihydroindeno[2*,*1-b]pyrrol-8(1H)-one*.** ^1^H NMR: δ 9.45 (1H, *br m*, NH), 7.84–7.76 (4H, *m*, ArH and OH), 7.19–7.12 (5H, *m*, ArH), 6.85 (1H, *s*, OH), 5.36 (1H, *d*, ^2^*J*_AX_ = 16.0, CH_2_), 5.13 (1H, *d*, ^2^*J*_AX_ = 16.0, CH_2_), 3.54 (3H, *s*, CH_3_), 2.55 (3H, s, NCH_3_).

**5c:** White solid; m.p 210–212°C; IR (KBr): 3413 (OH), 3337 (OH), 3241 (NH), 1736 (C = O), 1621 (NC = C), 1545 and 1345 cm^-1^ (NO_2_). MS: *m/z* (%) = 388 (2) [M-NO_2_]^+^, 172 (4), 149 (4), 124.1 (16), 123.1 (23), 98 (19), 95.1 (16), 81 (22), 80 (100), 65.1 (8), 64 (39), 50.1 (6).

**Major isomer (89%): *2-((4-fluorobenzyl)amino)-3a*,*8b-dihydroxy-1-methyl-3-nitro-3a*,*8b-dihydroindeno[1*,*2-b]pyrrol-4(1H)-one*.** ^1^H NMR: δ ppm, δ 9.86 (1H, *t*,^3^*J*_HH_ = 6.0, NH), 7.90–7.50 (4H, *m*, ArH), 7.40–6.90 (5H, *m*, ArH and OH), 6.37 (1H, *s*, OH),4.88–4.68 (2H, ABX, *m*, NCH_2_), 3.38 (3H, s, NCH_3_). ^13^C NMR:δ ppm, 29.1 (N-CH_3_), 45.5 (N-CH_2_), 80.9 (C-OH), 93.8 (C-OH), 115.3 (C-NO_2_),115.5,123.2,124.2,128.4,128.5,130.6,133.7,134.6,135.5,146.9 (Ar), 154.0 (= C-NCH2), 196.1 (C = O).

***Minor isomer (11%)*: *2-((4-fluorobenzyl)amino)-3a*,*8a-dihydroxy-1-methyl-3-nitro-3a*,*8a-dihydroindeno[2*,*1-b]pyrrol-8(1H)-one*.** ^1^H NMR: δ 9.46 (1H, *br m*, NH), 7.84–7.72 (5H, *m*, ArH and OH), 7.60 (2H, *m*, ArH), 7.39–7.31 (2H, *m*, ArH), 6.51 (1H, *s*, OH), 5.42 (1H, *d*, ^2^*J*_AX_ = 16.0, CH_2_), 5.21 (1H, *d*, ^2^*J*_AX_ = 16.0, CH_2_), 2.55 (3H, s, NCH_3_).

**5d:** White solid; m.p 96–98°C; IR (KBr): 3494 (OH), 3353 (OH), 3214 (NH), 1730 (C = O), 1639 (NC = C), 1566 and 1388 cm^-1^ (NO_2_). MS: *m/z* (%) = 354.1 (11) [M-NO_2_]^+^, 319.1 (39), 172.1 (54), 160.1 (23), 127.1 (35), 125.1 (100), 104.1 (56), 89.1 (25), 76.1 (45), 50.1 (18).

**Major isomer (95%): *2-((2-chlorobenzyl)amino)-3a*,*8b-dihydroxy-1-methyl-3-nitro-3a*,*8b-dihydroindeno[1*,*2-b]pyrrol-4(1H)-one*.** ^1^H NMR: δ ppm, δ 9.85 (1H, *t*,^3^*J*_HH_ = 6.0, NH), 7.81–7.66 (4H, *m*, ArH), 7.34–7.14 (5H, *m*, ArH and OH), 6.39 (1H, *s*, OH),4.90–4.84 (2H, ABX, *m*, NCH_2_), 3.37 (3H, s, NCH_3_). ^13^C NMR: δ ppm, 28.9 (N-CH_3_), 44.6 (N-CH_2_), 80.1 (C-OH), 92.9 (C-OH), 107.6 (C-NO_2_),123.2,124.3,127.5,128.5,129.4,129.5,130.6,131.4,133.7,135.5,135.5,146.8 (Ar), 154.0 (= C-NCH2), 195.0 (C = O).

**Minor isomer (5%): *2-((2-chlorobenzyl)amino)-3a*,*8a-dihydroxy-1-methyl-3-nitro-3a*,*8a-dihydroindeno[2*,*1-b]pyrrol-8(1H)-one*.** ^1^H NMR: δ 9.57 (1H, *br m*, NH), 7.64–7.58 (5H, *m*, ArH and OH), 7.56–7.50 (1H, *m*, ArH), 7.47–7.43 (3H, *m*, ArH), 6.53 (1H, *s*, OH), 5.40 (1H, *d*, ^2^*J*_AX_ = 16.0, CH_2_), 5.26 (1H, *d*, ^2^*J*_AX_ = 16.0, CH_2_), 2.51 (3H, s, NCH_3_).

**5e:** White solid; m.p 96–98°C; IR (KBr): 3472 (OH), 3378 (OH), 3258 (NH), 1730 (C = O), 1617 (NC = C), 1559 and 1385 cm^-1^ (NO_2_). MS: *m/z* (%) = 320.2 (9) [M-NO_2_]^+^, 161.2 (35), 160.1 (19), 132.1 (35), 106.1 (32), 104.1 (63), 91.1 (100), 76.1(45),65.1(14),56.1(12).

***Major isomer (93%)*: *3a*,*8b-dihydroxy-2-((4-methoxybenzyl)amino)-1-methyl-3-nitro-3a*,*8b-dihydroindeno[1*,*2-b]pyrrol-4(1H)-one*.**^1^H NMR: δ ppm, δ 9.80 (1H, *t*,^3^*J*_HH_ = 8.0, NH), 7.75–7.58 (5H, *m*, ArH and OH), 7.10 (2H, *d*, ^3^*J*_*HH*_ = 8.0, ArH), 6.82 (2H, *d*, ^3^*J* = 8.0, ArH), 6.36 (1H, *s*, OH),4.81–4.71 (ABX, 2H, *m*, NCH_2_), 3.77 (3H, *s*, CH_3_), 3.42 (3H, s, NCH_3_). ^13^C NMR: δ ppm, 29.6 (N-CH_3_), 46.3 (N-CH_2_), 55.5 (OCH_3_), 80.5 (C-OH), 93.4 (C-OH), 108.1 (C-NO_2_),114.5,123.7,124.8, 128.4, 130.5,131.1,134.2,136.1,147.4,154.3 (Ar), 159.0 (= C-NCH2), 195.6 (C = O).

***Minor isomer (7%)*: *3a*,*8a-dihydroxy-2-((4-methoxybenzyl)amino)-1-methyl-3-nitro-3a*,*8a-dihydroindeno[2*,*1-b]pyrrol-8(1H)-one*.**^1^H NMR: δ 9.45 (1H, *br m*, NH), 7.24–7.17 (7H, *m*, ArH and OH), 6.95–6.90 (2H, *d*, ^3^*J*_*HH*_ = 8.0, ArH), 6.62 (1H, *s*, OH), 5.30 (1H, *d*, ^2^*J*_AX_ = 16.0, CH_2_), 5.10 (1H, *d*, ^2^*J*_AX_ = 16.0, CH_2_), 3.75 (3H, *s*, CH_3_), 2.55 (3H, s, NCH_3_).

**5f:** White solid; m.p 199–200°C; MS:*m/z* (%) = 320.2 (9) [M-NO_2_]^+^, 161.2 (35), 160.1 (19), 132.1 (35), 106.1 (32), 104.1 (63), 91.1 (100), 76.1 (45), 65.1 (14), 56.1 (12).

**Major isomer (92%): *2-(benzylamino)-3a*,*8b-dihydroxy-1-methyl-3-nitro-3a*,*8b-dihydroindeno[1*,*2-b]pyrrol-4(1H)-one*.** ^1^H NMR: δ ppm, 9.42 (1H, *t*, ^3^*J*_HH_ = 6.0,NH), 7.90–6.90 (10 H, *m*, ArH and OH), 6.37 (1H, *s*, OH), 5.04–4.71 (ABX, 2H, *m*, NCH_2_), 3.39 (3H, s, NCH_3_). ^13^C NMR: δ ppm, 29.0 (N-CH_3_), 46.1 (N-CH_2_), 85.8 (C-OH), 92.8 (C-OH), 121.5 (C-NO_2_),, 123.3,124.2,126.3,127.6,129.7,130.8,133.4,135.9,137.9,146.5 (Ar), 153.0 (= C-NCH2), 195.2 (C = O).

**Minor isomer (8%): *2-(benzylamino)-3a*,*8a-dihydroxy-1-methyl-3-nitro-3a*,*8a-dihydroindeno[2*,*1-b]pyrrol-8(1H)-one***. ^1^H NMR: δ 9.47 (1H, *br m*, NH), 7.86–7.75 (5H, *m*, ArH and OH), 7.38–7.30 (5H, *m*, ArH), 7.34 (2H, *d*, ^3^*J* = 8.0, ArH), 6.44 (1H, *s*, OH), 5.42 (1H, *d*, ^2^*J*_AX_ = 16.0, CH_2_), 5.19 (1H, *d*, ^2^*J*_AX_ = 16.0, CH_2_), 2.55 (3H, s, NCH_3_).

**5g:** White solid; m.p 225–227°C.

**Major isomer (92%): *3a*,*8b-dihydroxy-1-methyl-3-nitro-2-((4-(trifluoromethyl)benzyl)amino)-3a*,*8b-dihydroindeno[1*,*2-b]pyrrol-4(1H)-one*.** ^1^H NMR: δ ppm, 9.95 (1H, *t*, ^3^*J*_HH_ = 6,NH), 7.66–7.51 (6H, *m*, ArH), 7.36–7.32 (2H, *d*, ^3^*J*_HH_ = 8.0,ArH), 7.23 (1H, *s*, OH), 6.39 (1H, *s*, OH), 4.96–4.88 (ABX, 2H, *m*, NCH_2_), 3.34 (3H, *s*, NCH_3_). ^13^C NMR: δ ppm, 29.0 (N-CH_3_), 45.8(N-CH_2_), 80.1 (C-OH), 92.8 (C-OH), 100.9 (C-NO_2_),123.1(CF_3_),124.1,125.4,125.5,126.9,130.5,133.6,135.4,143.5,146.8,154.2 (Ar), 158.8 (= C-NCH2), 195.1 (C = O).

**Minor isomer (8%): *3a*,*8a-dihydroxy-1-methyl-3-nitro-2-((4-(trifluoromethyl)benzyl)amino)-3a*,*8a-dihydroindeno[2*,*1-b]pyrrol-8(1H)-one*.** ^1^H NMR: δ 9.45 (1H, *br m*, NH),, 7.91–7.55(2H, *m*, ArH), 7.74–7.64(7H, *m*, ArH and OH), 5.50(1H, *d*, ^2^*J*_AX_ = 16.0, CH_2_), 5.32 (1H, *d*, ^2^*J*_AX_ = 16.0, CH_2_), 2.52 (3H, s, NCH_3_).

**5h:** White solid; m.p 188–190°C.

**Major isomer (94%): *2-(ethylamino)-3a*,*8b-dihydroxy-1-methyl-3-nitro-3a*,*8b-dihydroindeno[1*,*2-b]pyrrol-4(1H)-one*.** ^1^H NMR: δ ppm, 9.95 (1H, *t*, ^3^*J*_HH_ = 6,NH), 7.86–7.63 (4H, *m*, ArH), 7.21 (1H, *s*, OH), 6.35 (1H, *s*, OH), 3.72–3.54 (ABX, 2H, *m*, NCH_2_), 3.48 (3H, *s*, NCH_3_), 1.15–1.09 (3H, *t*, ^3^*J*_HH_ = 6,CH_3_). ^13^C NMR: δ ppm, 15.8 (CH_3_), 29.2 (N-CH_3_),37.9(CH_2_), 80.0 (C-OH), 92.9 (C-OH), 107.2 (C-NO_2_),123.3,124.4,130.7,133.8,136.7,147.0 (Ar), 153.2 (= C-NCH2), 195.1 (C = O).

**Minor isomer (6%):*2-(ethylamino)-3a*,*8a-dihydroxy-1-methyl-3-nitro-3a*,*8a-dihydroindeno[2*,*1-b]pyrrol-8(1H)-one*.** ^1^H NMR: δ 7.64–7.59 (5H, *m*, ArH and OH), 6.58 (1H, *s*, OH), 4.21–3.76 (ABX, 2H, *m*, NCH_2_), 3.40 (3H, *s*, NCH_3_).

**5i:** White solid; m.p 130–132°C.

**Major isomer (96%): *2-((2-fluorobenzyl)amino)-3a*,*8b-dihydroxy-1-methyl-3-nitro-3a*,*8b-dihydroindeno[1*,*2-b]pyrrol-4(1H)-one*.** ^1^H NMR: δ ppm, 9.89 (1H, *t*, ^3^*J*_HH_ = 6, NH), 7.80–7.59 (5H, *m*, ArH and OH), 7.38–7.06 (4H, *m*, ArH), 6.39 (1H, *s*, OH), 4.83–4.74 (ABX, 2H, *m*, NCH_2_), 3.42 (3H, *s*, NCH_3_). ^13^C NMR: δ ppm, 28.9 (N-CH_3_), 40.76(N-CH_2_), 80.1 (C-OH), 93.0 (C-OH), 107.6 (C-NO_2_),115.5,123.2,124.3,124.7,125.1,128.5,129.8,130.6,133.7,135.6,146.8,153.9, (Ar), 160.5 (= C-NCH2), 195.0 (C = O).

**Minor isomer (4%): *2-((2-fluorobenzyl)amino)-3a*,*8a-dihydroxy-1-methyl-3-nitro-3a*,*8a-dihydroindeno[2*,*1-b]pyrrol-8(1H)-one***. ^1^H NMR: δ 9.45 (1H, *br m*, NH), 7.97–7.77(5H, *m*, ArH and OH), 7.38–7.35 (4H, *m*, ArH), 5.38 (1H, *d*, ^2^*J*_AX_ = 16.0, CH_2_), 5.29 (1H, *d*, ^2^*J*_AX_ = 16.0, CH_2_), 2.52 (3H, s, NCH_3_).

### Computational studies

#### Density functional theory (DFT)

DFT is an effective method for determining electron density and energy parameters in compounds. Its application extends to the determination of atomic, molecular, crystalline, and surface structures and elucidates their interplay. These calculations are carried out with the Gaussian 09W software [22]. Using the B3LYP method coupled with a 6–31++G(d,p) basis set, the wavenumbers associated with the vibrations were carefully calculated. The B3LYP functional is widely recognized for its ability to accurately describe harmonic vibrational properties in molecules of modest to moderate size. The positive values of IR frequencies derived from this method imply that the optimized structure has a minimum on the potential energy surface. The generated check files were analyzed using GuassView 6.0.

#### Molecular docking studies

Molecular docking analysis investigated the interaction between the synthesized compounds and the SARS-CoV-2 M^pro^ protein using Schrodinger’s Maestro Molecular Modeling platform. The 3D structure of the protein (PDB: 6W63) [23] was used. All protein preparation for docking was performed, including the use of Protein Preparation Wizard [24] to modify missing residues and ensure structural integrity. GuassView 6.0 helped to visualize the structures of the compounds, which were subsequently converted to.pdb files for the ligprep module. For ligand preparation, the OPLS_2005 force field was used at a pH of 7.0±2 [25]. The individual binding sites were defined by a 26 Å grid array created with Glide. Standard accuracy and flexible ligand sampling were used, with the analysis showing 10 poses per ligand.

#### Molecular dynamic simulation

A molecular dynamics (MD) simulation was performed with Desmond using the Schroedinger-Maestro interface [26]. The results are from the MD simulation performed for the complex following the previous docking phase. An orthorhombic cell filled with water molecules was used, which is representative of the SPC model. Sufficient Na ions were introduced into the system to neutralize the total charge of the complex. The simulation lasted 100 ns and used the NPT ensemble, keeping the number of atoms, the pressure of 1.01325 bar, and the temperature of 300 K constant. The Nose-Hoover chain method with an interval of 1.0 ps was used as the standard thermostat, and the Martyna-Tobias-Klein method with an interval of 2.0 ps was used as the barostat. The interaction diagram of the Maestro simulation was used to analyze the molecular dynamic simulation.

## Supporting information

S1 File (DOCX)
