# Supplementary material for: Synthesis, molecular docking analysis, molecular dynamic simulation, ADMET, DFT, and drug likeness studies: Novel Indeno[1,2-b]pyrrol-4(1H)-one as SARS-CoV-2 main protease inhibitors
Source: PLoS One. 2024 Mar 22;19(3):e0299301. doi: 10.1371/journal.pone.0299301 (PMC10959350; doi:10.1371/journal.pone.0299301)
Supplement: S1 File — (DOCX) [file pone.0299301.s001.docx]

**Supporting Information**

**Synthesis, Molecular Docking Analysis, Molecular Dynamic Simulation, ADMET, DFT, and Drug Likeness Studies: Novel Indeno[1,2-*b*]pyrrol-4(1*H*)-one as SARS-CoV-2 main protease inhibitors**

Davood Gheidari *^a^ Morteza Mehrdad ^a^ Mohammad Bayat *^b^

*^a^Department of Chemistry, Faculty of Science, University of Guilan, Rasht, Iran*

*^b^Department of Chemistry, Faculty of Science, Imam Khomeini International University, Qazvin, Iran*

*Correspondence: Mohammad Bayat, [bayat_mo@yahoo.com](mailto:bayat_mo@yahoo.com); [m.bayat@sci.ikiu.ac.ir](mailto:m.bayat@sci.ikiu.ac.ir);Davood Gheidari, [davoodgheidari@phd.guilan.ac.ir](mailto:davoodgheidari@phd.guilan.ac.ir) ; [davoodgheidari@gmail.com](mailto:davoodgheidari@gmail.com)

**The Table of Contents**

| **Title** | **Page** |
| --- | --- |
| Title, author’s name, address and table of contents | 1 |
| Experimental Section; General remarks | 2 |
| **Figure 1.** Molecular structures of products **5a–i**. | 3 |
| **Figure 2.** Optimized structures of the compounds **5a–i**. | 4 |
| **Figure 3.** FMOs of the compounds **5a–i**. | 5-7 |
| ^1^H and ^13^C NMR and IR and Mass spectrums of **5a** | 8-11 |
| ^1^H and ^13^C NMR and IR and Mass spectrums of **5b** | 12-15 |
| ^1^H and ^13^C NMR and IR and Mass spectrums of **5c** | 16-19 |
| ^1^H and ^13^C NMR and IR and Mass spectrums of **5d** | 20-23 |
| ^1^H and ^13^C NMR and IR spectrums of **5e** | 24-27 |
| ^1^H and ^13^C NMR and IR and Mass spectrums of **5f** | 28-30 |
| ^1^H and ^13^C NMR and IR spectrums of **5g** | 31-32 |
| ^1^H and ^13^C NMR and IR spectrums of **5h** | 33-34 |
| ^1^H and ^13^C NMR and IR spectrums of **5i** | 35-36 |

**Experimental Section**

**General remarks:**

Melting points were measured on an Electrothermal 9100 apparatus. Mass spectra were recorded with an Agilent 5975C VL MSD with Triple-Axis Detector operating at an ionization potential of 70 Ev. ^1^H and ^13^C NMR spectra were measured (DMSO) with a Bruker Bio Spin spectrometer at 400 and 100 MHz, respectively. IR spectra were recorded on a Bruker Tensor 27, ῡ in cm^-1^. All NMR spectra at room temperature were determined in DMSO-*d*_6_. Chemical shifts are reported in parts per million (*δ*) downfield from an internal tetramethylsilane reference. Coupling constants (*J* values) are reported in hertz (Hz), and spin multiplicities are indicated by the following symbols: s (singlet), d (doublet), t (triplet), q (quartet), m (multiplet). All chemicals were purchased from Merck or Aldrich and were used without further purification.

* Signals related to residual ethanol, are indicated on the spectra.

|  |  |  |
| --- | --- | --- |
|  |  |  |
|  |  |  |

**Figure 1.** Molecular structures of products **5a–i**.

| 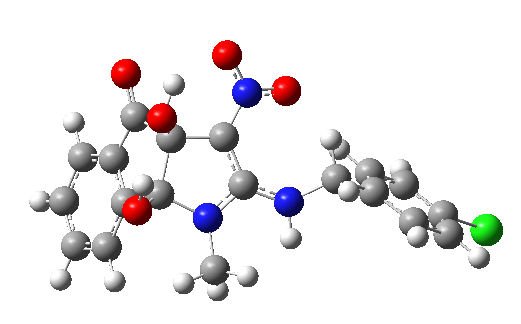  **5a** | 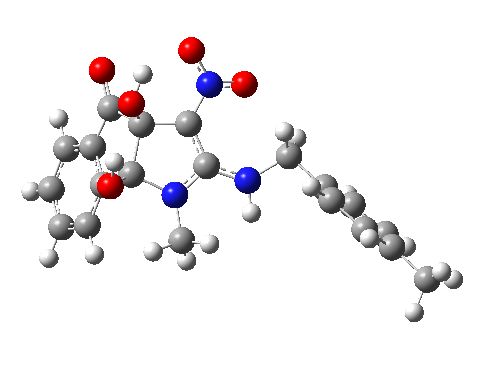  **5b** | 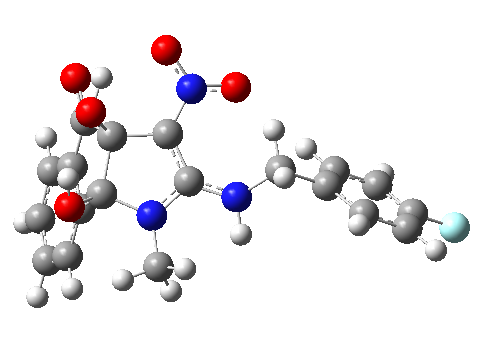  **5c** |
| --- | --- | --- |
| 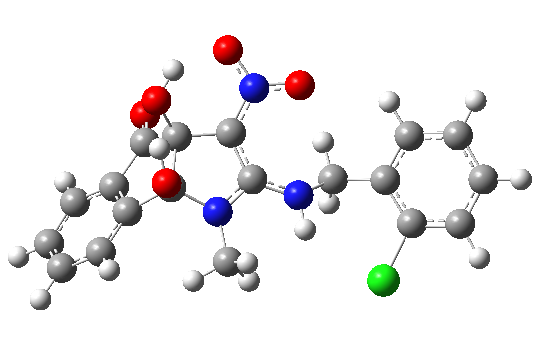  **5d** | 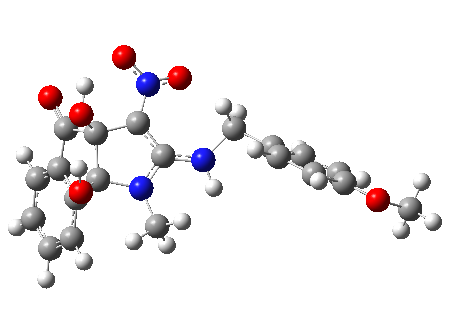  **5e** | 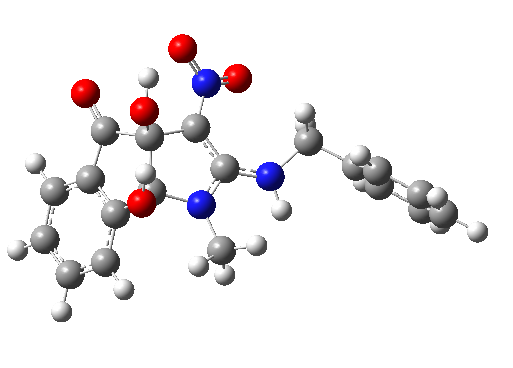  **5f** |
| 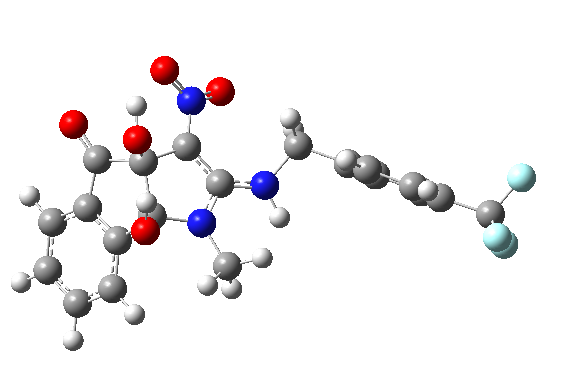  **5g** | 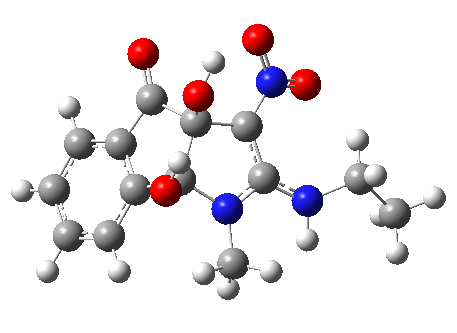  **5h** | 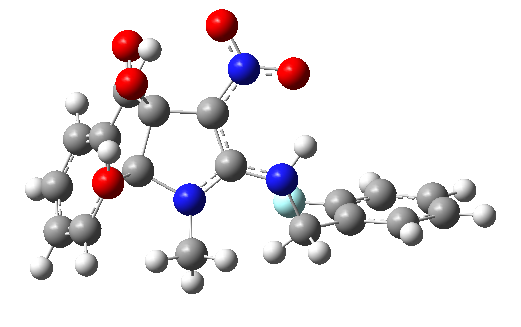  **5i** |
| **Figure 2.** Optimized structures of the compounds **5a–i**. | | |

|  | **HOMO** | **LUMO** |
| --- | --- | --- |
| **5a** | 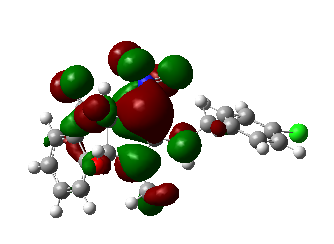 | 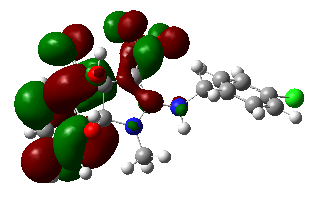 |
| **5b** | 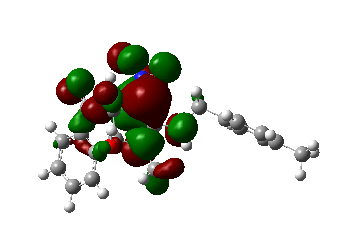 | 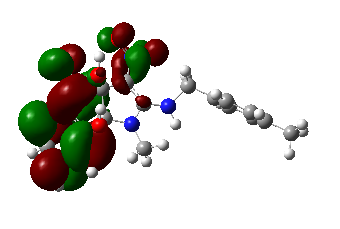 |
| **5c** | 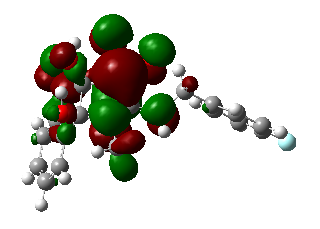 | 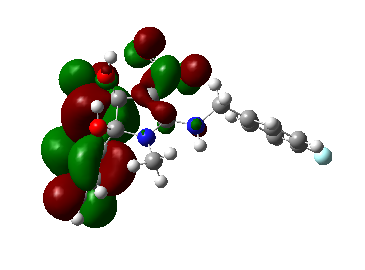 |
| **5d** | 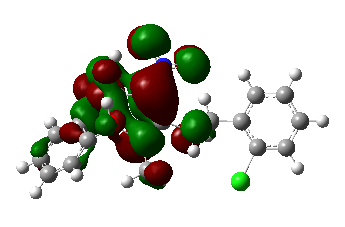 | 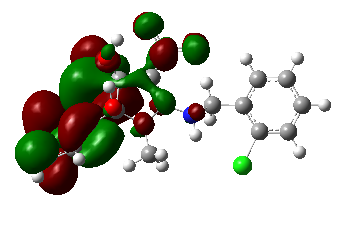 |
| **5e** | 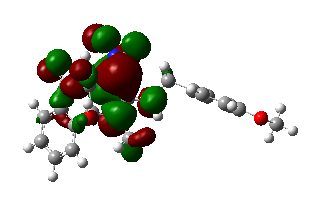 | 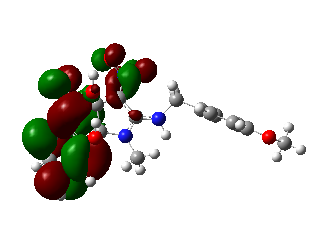 |
| **5f** | 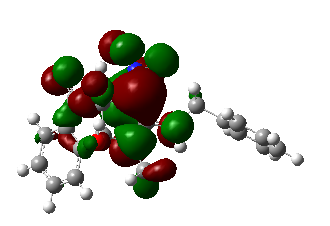 | 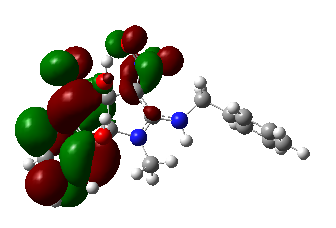 |
| **5g** | 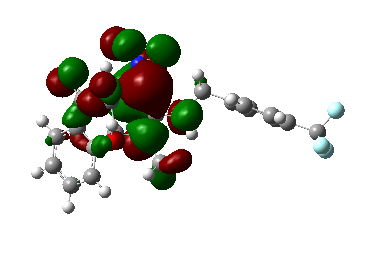 | 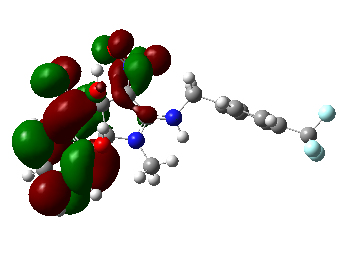 |
| **5h** | 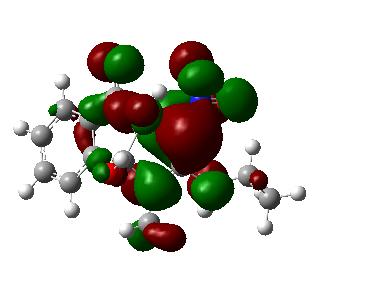 | 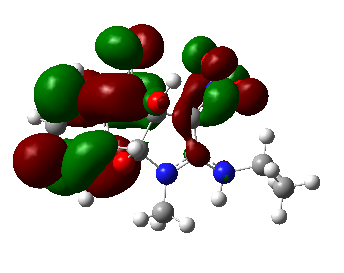 |
| **5i** | 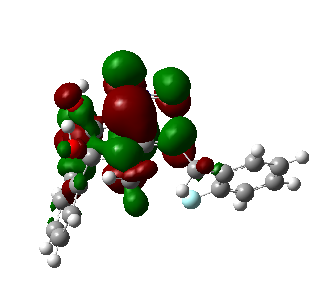 | 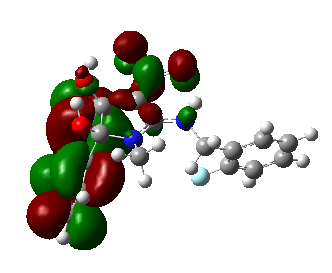 |

**Figure 3.** FMOs of the compounds **5a–i**.


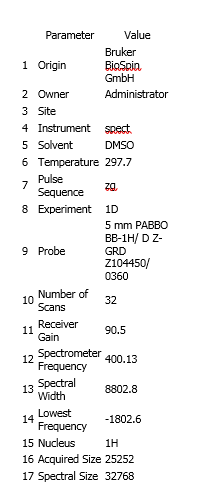

**^1^H NMR of 5a**

**
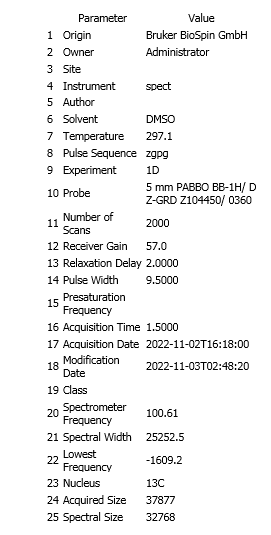
**

**^13^C NMR of 5a**

**
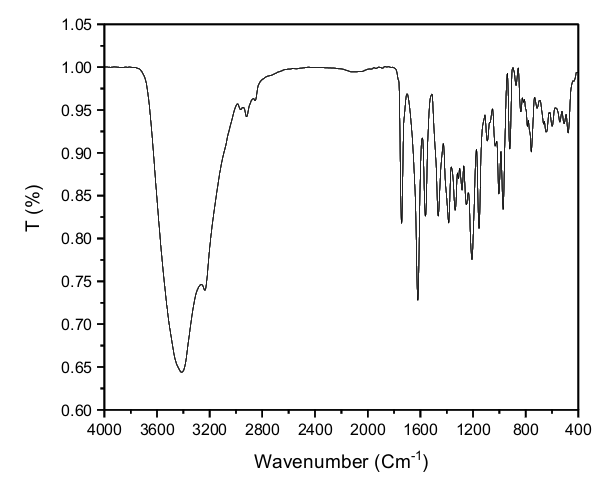
**

**IR of 5a**

**
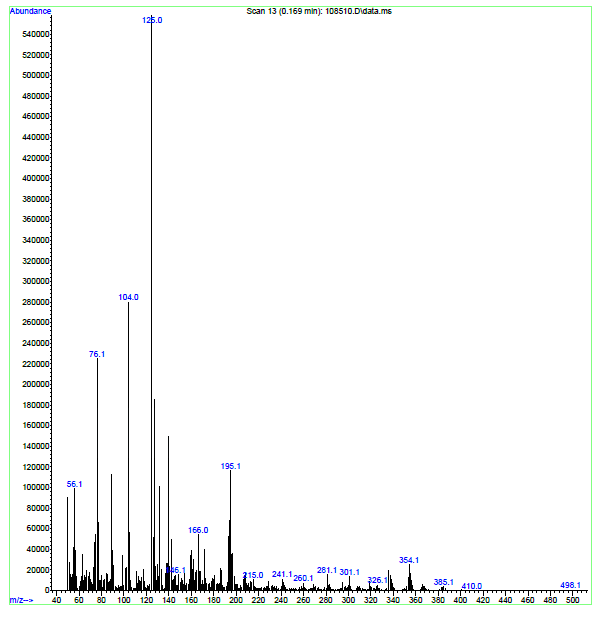
**

**MS of 5a**


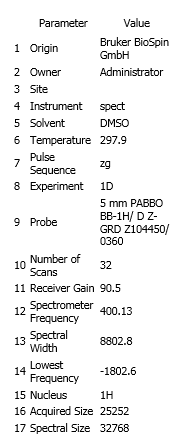

**^1^H NMR of 5b**


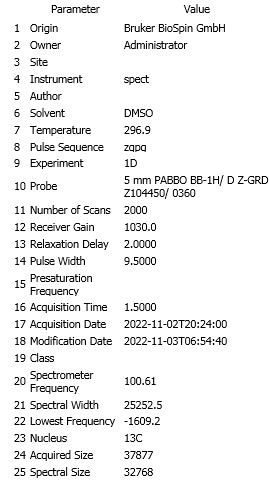


**^13^C NMR of 5b**

**
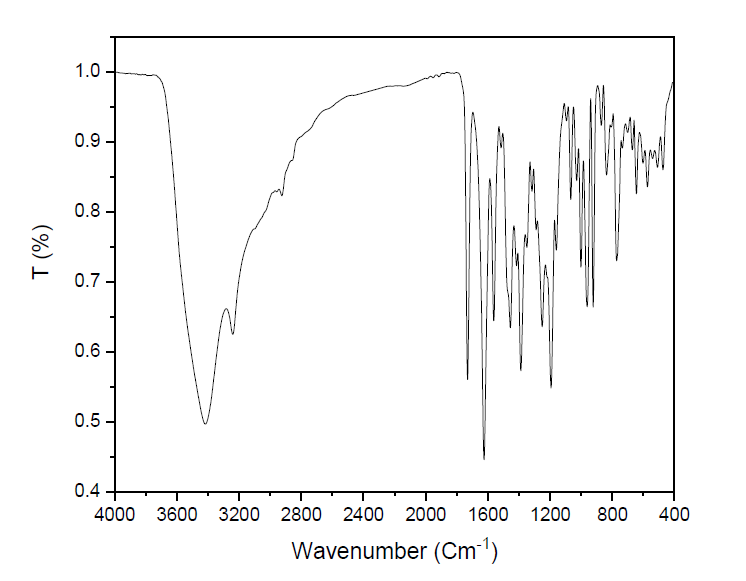
**

**IR of 5b**

**
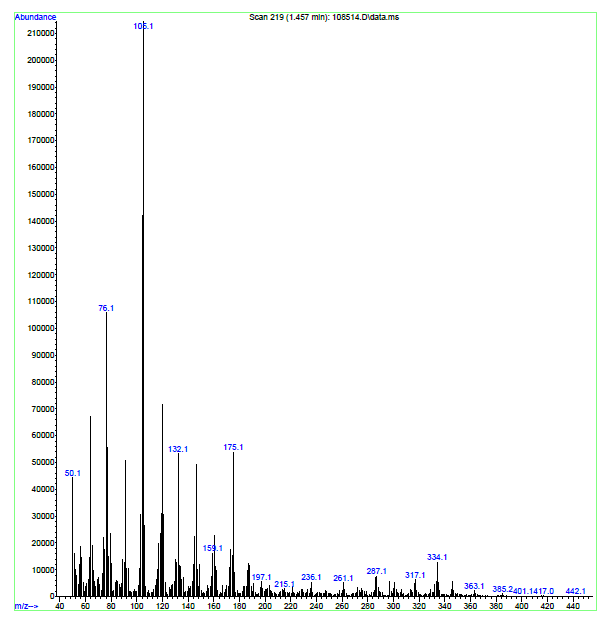
**

**MS of 5b**

**
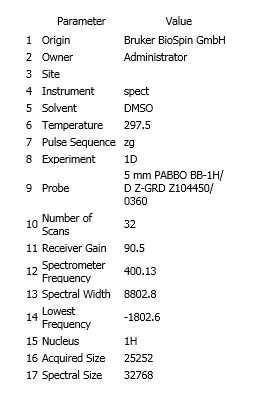
**

**H NMR of 5c**

**
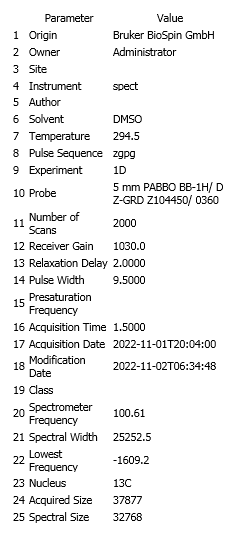
**

**^13^C NMR of 5c**

**
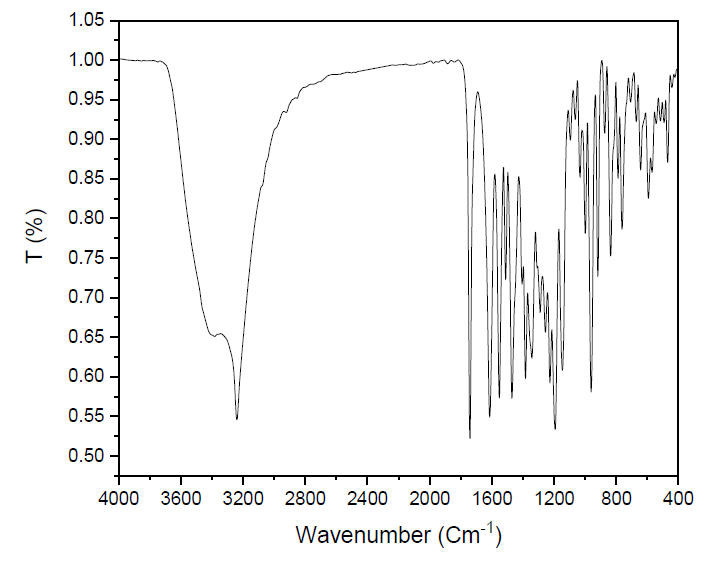
**

**IR of 5c**


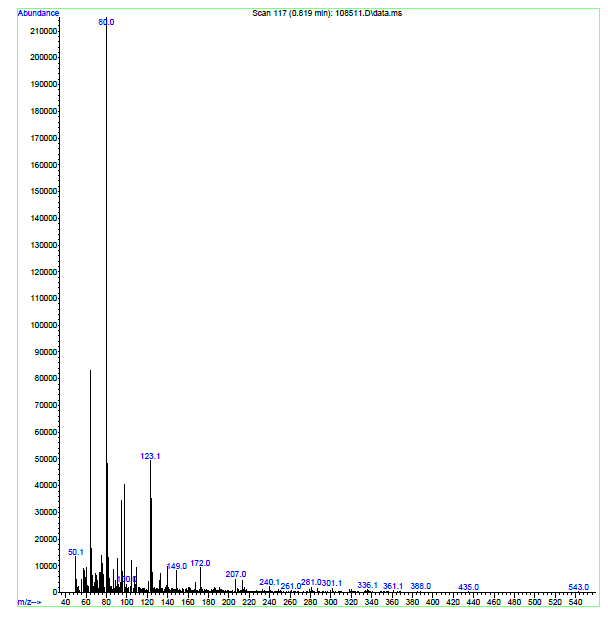


**MS of 5c**


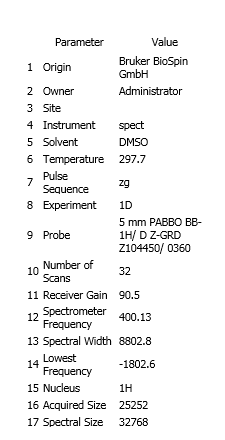

EtOH

EtOH

**^1^H NMR of 5d**


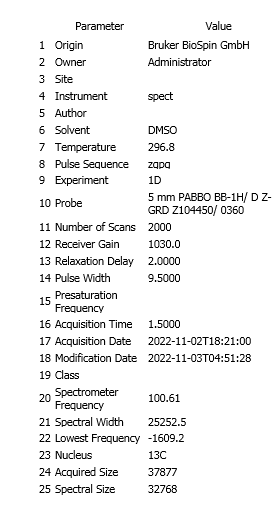


**^^**

EtOH

EtOH

**^13^C NMR of 5d**


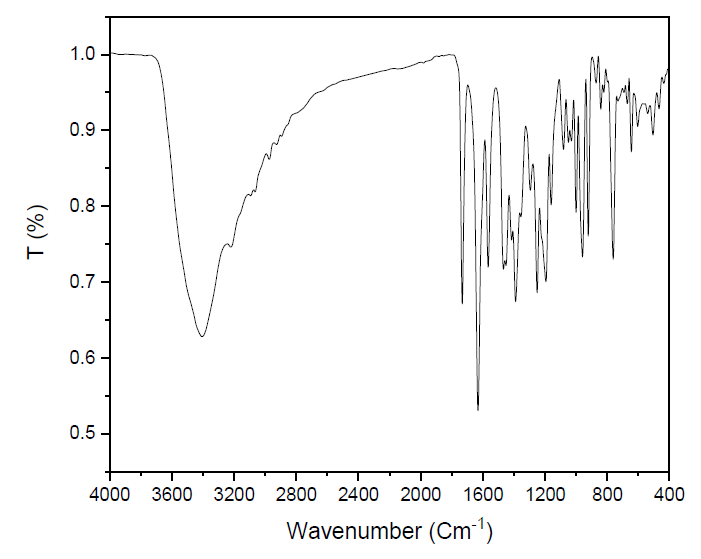


**IR of 5d**


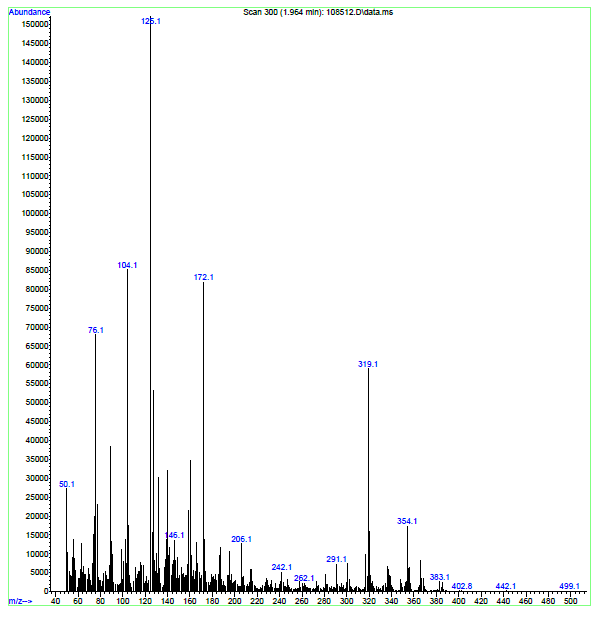


**MS of 5d**

**
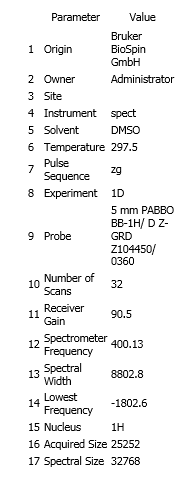
**

EtOH

EtOH

**^1^H NMR of 5e**

**
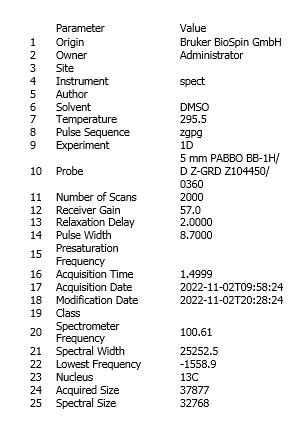
**

EtOH

EtOH

**^13^C NMR of 5e**


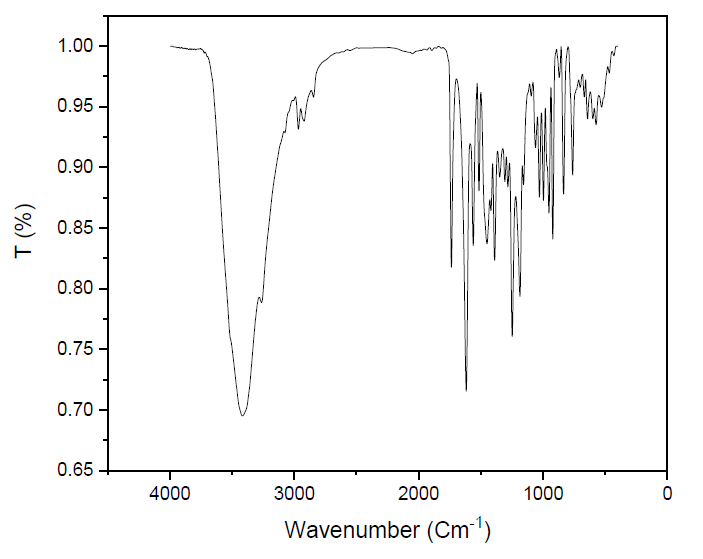


**IR of 5e**


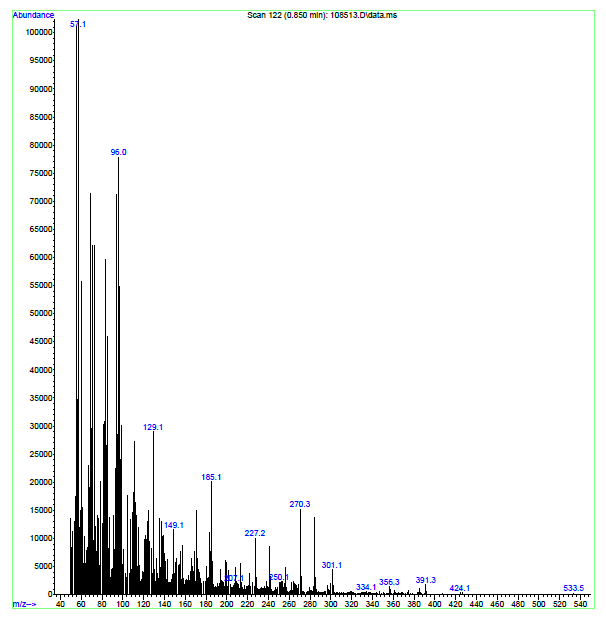


**MS of 5e**


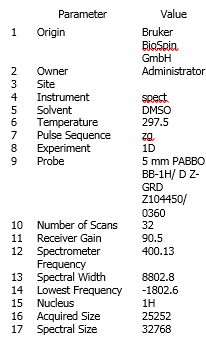


**^1^H NMR of 5f**


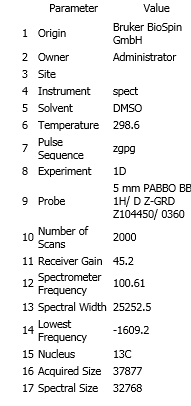

**^13^C NMR of 5f**


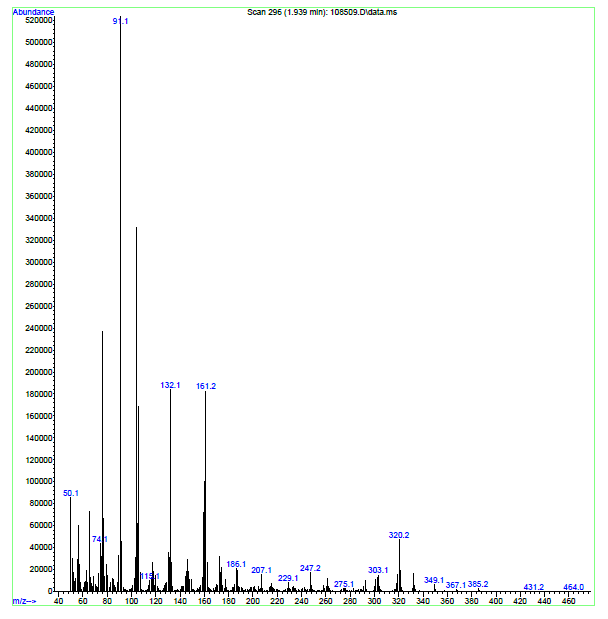


**MS of 5f**

**
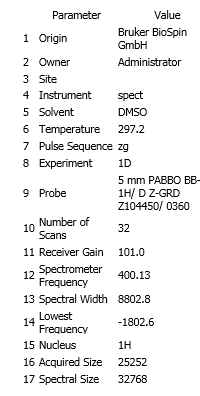
**

**^1^H NMR of 5g**


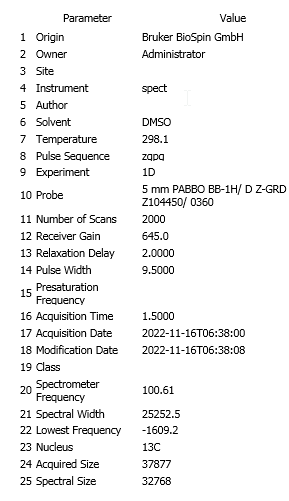

**^13^C NMR of 5g**

**
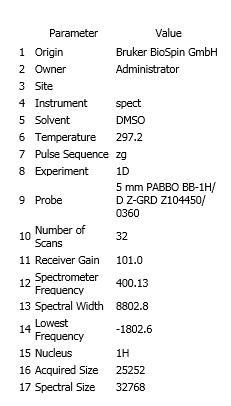
**

**^1^H NMR of 5h**

**
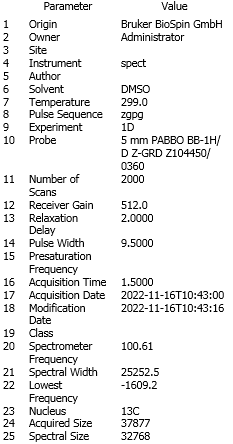
**

**^13^C NMR of 5h**

**
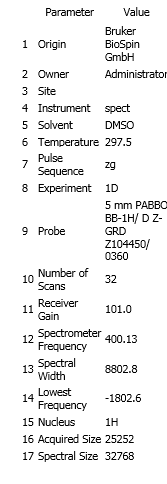
**

EtOH

EtOH

EtOH

**^1^H NMR of 5i**


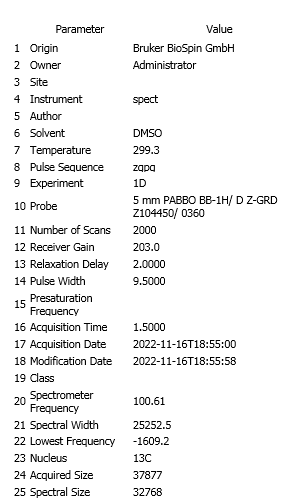

 **^13^C NMR of 5i**

EtOH

EtOH
